# On the Design and 3D Printing of Thin Stochastic Porous Structures Made of PLA

**DOI:** 10.3390/polym18212708

**Published:** 2026-11-01

**Authors:** Kazuki Sasaki, Sharifu Ura

**Affiliations:** 1 Graduate School of Engineering, Kitami Institute of Technology, 165 Koen-cho, Kitami 090-8507, Japan; 2 Division of Mechanical and Electrical Engineering, Kitami Institute of Technology, 165 Koen-cho, Kitami 090-8507, Japan; 3 Faculty of Engineering, University of the Ryukyus, 1 Senbaru, Nishihara 903-0213, Japan

**Keywords:** polylactic acid, additive manufacturing, fused filament fabrication, porous structure, stochastic porosity

## Abstract

Thin stochastic porous structures have potential applications as flexible structural components and functional elements in heat and mass transfer systems, filtration and separation membranes, tissue engineering scaffolds, and lightweight structures requiring controlled porosity and permeability. However, accurately designing and fabricating these structures using affordable additive manufacturing technologies remains challenging. This study presents a computer-aided design (CAD) method for generating thin stochastic porous structures and evaluates their printability in polylactic acid (PLA) using fused filament fabrication (FFF). The method divides a planar region into cells and generates polygonal pores with random sizes, shapes, and orientations, while avoiding disconnected regions and excessively thin walls. An analytical formulation calculates porosity, and a sensitivity analysis identifies key geometric parameters. Sixteen specimens were fabricated using a commercially available, affordable 3D printer across four configurations with varying structural and layer thicknesses and deposition passes. The structures were evaluated for pore geometry, porosity, and thickness accuracy. Of the configurations tested, a designed thickness of 200μm fabricated with two deposition passes of 100μm each achieved the highest average intersection over union (87.56%), the highest average $F_{1}$ score (93.36%), the lowest average relative porosity error (2.38%), and the smallest relative thickness deviation (approximately 15%). These results show that thin stochastic porous PLA structures can be fabricated using a commercially available FFF printer. The findings also underscore the importance of selecting appropriate layer thicknesses and deposition strategies to improve geometric fidelity.

## 1. Introduction

Additive manufacturing (AM) enables the direct production of components from three-dimensional digital models through the successive addition of material [1,2]. Compared with conventional subtractive and formative processes, AM can reduce the need for specialized tools, fixtures, and intermediate processing steps. It can also reduce material waste, accommodate design modifications rapidly, and fabricate components with complex internal geometries [3].

Among the various AM processes, fused filament fabrication (FFF), also known as fused deposition modeling (FDM) [2,4] is widely used because of its straightforward operation, relatively low equipment cost, and compatibility with various materials [2,5].

Polylactic acid (PLA) is considered one of the most suitable thermoplastics for fused filament fabrication (FFF) due to its low thermal shrinkage, good dimensional stability, and relatively high stiffness [6,7,8]. These properties reduce the risk of warping and support the production of geometrically stable components. Commercial PLA filaments are commonly processed at nozzle temperatures of approximately 190–220 °C. A heated build chamber is generally unnecessary [2,8]. PLA can also be produced from renewable biological resources [6,7]. Tests of desktop printers have reported lower total volatile organic compound emissions from PLA than from Acrylonitrile Butadiene Styrene (ABS) under selected operating conditions. However, the emission profile depends on the filament formulation and process conditions [9]. These characteristics make PLA suitable for prototypes, customized products, and functional components with complex internal and external geometries. Furthermore, the properties of FFF-printed PLA components can be adjusted through infill density, infill geometry, and thermal treatment [10,11,12].

Porous structures have been extensively studied in the literature from multiple perspectives, including structural design, functional performance, and manufacturing feasibility. For example, Fang et al. [13] demonstrated the effectiveness of porous structures as a design concept for developing load-bearing components with improved structural performance. Their proposed geometric optimization method enables control of pore shape, size, and orientation while reducing the number of design variables and improving damage tolerance.

From a functional perspective, Lee et al. [14] demonstrated the utility of stochastic porous structures for controlling and investigating fluid transport. They used Material Jetting, an additive manufacturing process that selectively deposits and UV-cures liquid photopolymer droplets, to fabricate three-dimensional porous micromodels that capture the internal characteristics of natural sandstone. Their results showed that stochastic reconstruction enables control over pore-scale geometric characteristics, including porosity and spatial correlation length, which influence permeability and fluid-flow behavior. The agreement between experimental measurements and numerical predictions established the suitability of these structures for investigating pore-scale transport phenomena.

Using a different additive manufacturing process, Patsoukis Dimou et al. [15] investigated the fabrication and fluid-flow performance of porous micromodels produced by stereolithography (SLA). Their study demonstrated that pore connectivity and manufacturing accuracy are strongly influenced by pore dimensions and printing conditions. Pore throats measuring 300–500 μm were fabricated reliably, whereas smaller dimensions exhibited increased manufacturing errors and channel blockage. In heterogeneous structures, pore throats below 140 μm experienced non-repeatable blockage. Notably, 200 μm pore throats could be fabricated using a 100 μm layer thickness but became blocked when the layer thickness was reduced to 25 μm. These findings indicate that printing resolution does not necessarily improve the fabrication of internal pore channels and that geometric design and manufacturing conditions must be considered together.

Bernadi-Forteza et al. [16] provided a broader perspective through a systematic review of the additive manufacturing of organic porous structures. Their review examined regular and stochastic porous architectures, geometric generation methods, and manufacturing technologies, particularly for biomedical scaffolds. The findings revealed that lattice-based structures remain predominant, while mathematically generated organic porous structures are increasingly being investigated. Powder bed fusion technologies were frequently employed to produce complex porous geometries, whereas material extrusion processes received comparatively limited attention. The review also identified unresolved challenges in generating self-supporting organic porous structures suitable for additive manufacturing.

Collectively, these studies demonstrate the significance of porous structures as both structural and functional elements. However, manufacturing approaches employed to investigate complex stochastic porous structures have predominantly relied on high-resolution additive manufacturing, with geometries investigated using more accessible materials. Fused filament fabrication (FFF) offers relatively affordable equipment and material costs, as well as straightforward processing requirements. Among the materials used in FFF, polylactic acid (PLA) has well studied material properties, and its sustainability properties (e.g., CO_2_ footprints) are also well studied [17]. In particular, polylactic acid (PLA) is a widely used FFF material suitable for investigating the manufacturability of complex porous geometries.

Although porous structures have been extensively studied, thin stochastic porous structures have received comparatively limited attention. These structures offer potential applications in flexible components, heat- and mass-transfer devices, and artificial membranes. Additionally, stacking thin porous layers with partially overlapping pores can yield effective openings smaller than the minimum feature size attainable through direct fabrication. For instance, stacking two layers with 1 mm pores and appropriate offsets can result in openings smaller than 1 mm. Nevertheless, fabrication limitations, such as insufficient resolution and pore closure, as well as design constraints, including disconnected shells and excessively thin walls, hinder the accurate reproduction of intended porous architectures.

To address these issues, this study introduces a computer-aided design (CAD) method for generating thin stochastic porous structures that avoid disconnected shells and excessively thin walls. The printability of the resulting designs is assessed by fabricating thin PLA specimens using FFF. The structure of this paper is as follows: Section 2 reviews previous research on additively manufactured porous structures, with particular emphasis on PLA-based FFF. Section 3 details with the proposed CAD method. Section 4 presents the fabrication results and quantitative comparisons between the designed and fabricated structures. Section 5 discusses how the proposed method can be extended to incorporate variations in pore geometry and nested porous structures, as well as the potential use of advanced filament materials beyond PLA. Section 6 concludes the paper.

## 2. Related Work

Numerous authors have researched the fabrication of porous structures through AM. To provide an overview of this research, this section reviews relevant studies with an emphasis on design methods, the use of PLA as a fabrication material, geometric accuracy, and potential applications.

Herath et al. [18] applied Voronoi tessellation to design biodegradable, patient-specific bone scaffolds with macropores for the surgical treatment of critical-sized bone defects. The scaffolds were fabricated from commercial-grade PLA by fused filament fabrication (FFF). The fabricated scaffolds had pore sizes ranging from 4.0 to 11.8mm, a porosity of 71%, and a mean geometric agreement of 76% between the as-designed and as-manufactured structures. Finite element analysis and compression tests also demonstrated the potential of Voronoi-based design and FFF for patient-specific bone scaffolds.

Ernst et al. [19] investigated the FFF fabrication of small porous structures with an Ultimaker 3. They examined PLA, carbon-nanotube-filled PLA, and titanium-dioxide-filled PVA, which served as a soluble support material for lattice-like scaffolds. The study evaluated the effects of build-plate calibration and the heights of the first three layers and particulate additives on print quality and individual line-width accuracy. The results showed that both process settings and material composition affect the accuracy and reproducibility of small porous structures. However, the study did not report a single numerical measure of overall accuracy.

Cao et al. [20] designed gyroid and other triply periodic minimal surface (TPMS) lattices with uniform and graded wall thicknesses for biomedical scaffolds and flexible structural applications. The lattices were fabricated using fused filament fabrication (FFF) with biodegradable PBAT/PLA composite filaments, which exhibited good printability. The gyroid lattices demonstrated higher load-bearing and energy-absorption capacities than the other TPMS lattices investigated. These findings suggest that spatial variations in pore geometry can influence mechanical performance and motivate further investigation of stochastic porous structures with variable pore sizes, shapes, and distributions.

Khan et al. [21] investigated the tensile properties of FFF-fabricated PLA prismatic cellular structures. A full factorial design and analysis of variance were used to evaluate the effects of cell size, wall thickness, and fillet radius. The triangular structures achieved a tensile strength of 24.28MPa, a Young’s modulus of 1.36GPa, and a strength-to-weight ratio of 5.78MPa/g. The results showed that cell size and wall thickness strongly affected the tensile properties and fracture behavior, demonstrating the importance of geometric control in the design of porous structures.

Navidpour et al. [22] fabricated PLA acoustic absorbers with uniform and functionally graded porous structures using FFF. The graded structures combined infill densities of 40/50/60% or 70/80/90%. Their sound-absorption coefficients were measured using an impedance tube. The reversed graded structure achieved a peak sound-absorption coefficient above 0.95 and provided effective absorption over a broad frequency range. These results demonstrate that spatial variations in porosity and structural orientation can influence acoustic performance. They also suggest the potential of superposition-based designs that combine pore patterns with different characteristic sizes or densities to achieve broadband sound absorption.

Boualleg et al. [23] developed a computational method for flexible stochastic lattices based on three-dimensional Voronoi tessellation. The method controlled the cell number, strut thickness, and relative density, while a minimum seed-point distance prevented excessively small cells. The structures were fabricated by photopolymerization and evaluated through compression tests and finite element analysis. Strong agreement was obtained for relative densities ranging from 6% to 18%. This constrained-randomness approach maintains stochastic geometry while eliminating features unsuitable for fabrication.

Cronau and Engstler [24] investigated the impact-energy absorption of stochastic Voronoi lattices by varying strut diameter and seed-point density, which affect relative density and mechanical response. Uniform and graded polyamide 12 lattices were fabricated by selective laser sintering and tested at 5m/s. A design-of-experiments analysis showed that 25% relative density, small strut diameters, and high strut densities favored energy absorption. Local gradation reduced the initial impact stress peak but decreased overall energy absorption, highlighting the trade-off between peak-load reduction and total energy absorption.

Wang et al. [25] developed PLA/graphene oxide (GO) porous scaffolds for bone-tissue engineering by optimizing material composition, pore geometry, and fused deposition modeling (FDM) parameters. An orthogonal experimental design was used to optimize the printing conditions. The addition of 0.1% GO increased the tensile and compressive moduli by 35.6% and 35.8%, respectively. Schwarz P and Gyroid triply periodic minimal surface (TPMS) scaffolds exhibited higher compressive strength than conventional grids due to reduced stress concentration and more uniform load distribution. They also promoted bone marrow stromal cell adhesion, proliferation, and osteogenic differentiation, demonstrating their potential for bone repair.

Jain and Gupta [26] reviewed the design and fabrication of FDM-printed PLA lattices, identifying PLA as a biodegradable thermoplastic suitable for strong lattice structures at relatively low processing temperatures. The review covered periodic geometries, including octet truss, body-centered cubic, and cubic close-packed lattices, alongside cell size, lattice dimensions, layer height, nozzle temperature, print speed, infill percentage, support type, and build orientation. Ambient temperature and humidity also affected mechanical strength and surface quality. These findings highlight the combined influence of geometry, fabrication parameters, and environmental conditions on the performance of PLA lattices.

Guessasma et al. [27] designed cellular PLA structures by sequentially introducing spherical voids into CAD models, with target porosities of 10% to 60%. The structures were fabricated by fused deposition modeling and evaluated using X-ray microtomography, compression tests with up to 80% height reduction, and microstructure-based finite element analysis. Reduced layer thickness minimized process-induced defects, mechanical anisotropy, and performance loss. Consequently, the compressive behavior depended primarily on the amount and spatial distribution of designed porosity.

González González et al. [28] proposed a multi-objective optimization framework for FDM-printed PLA scaffolds with Gyroid, Primitive, and Diamond TPMS architectures. A Box–Behnken design and response surface methodology evaluated the effects of layer thickness, extrusion temperature, and target porosity on mechanical performance and discrepancies between designed and fabricated volumes and porosities. The predictive models achieved $R^{2}>0.77$, with most exceeding 0.90, while 83% of validation measurements had relative errors below 10%. These findings highlight the need to consider both mechanical performance and geometric fidelity in porous structure design.

Vallejos Baier et al. [29] evaluated a low-cost, open-source FFF printer for PLA bone-regeneration scaffolds with physiologically relevant pore sizes of 100–500μm. Compression tests and finite element analysis assessed mechanical performance, while pore size and porosity were compared with CAD. The low-cost printer demonstrated superior mechanical performance and geometric resolution compared to the commercial counterpart. The findings further indicated that pore geometry influences the apparent elastic modulus, enabling control of scaffold properties through geometric design.

Gunes et al. [30] investigated the dimensional accuracy of FDM-printed PLA parts using digital caliper measurements, the Taguchi method, and analysis of variance. The authors reported an optimal parameter combination of concentric infill, 3mm wall thickness, 70% infill density, and 200μm layer thickness. An artificial neural network predicted dimensional accuracy with an average $R^{2}>0.95$ across the tested geometries. These results underscore the significant impact of printing parameters and part geometry on dimensional accuracy.

Myers et al. [31] optimized FDM parameters for PLA. The study focused on Schoen Gyroid and Schwarz Primitive orthopedic scaffolds using these optimized parameters. A full factorial analysis was conducted to evaluate the effects of layer height, print speed, and flow rate. The optimal settings for achieving geometric accuracy were 0.16mm for layer height, 50mm/s for print speed, and 90% for flow rate, respectively. At identical porosity and fabrication conditions, the Gyroid and Primitive scaffolds exhibited elastic moduli of 264 and 221MPa, and compressive stress limits of 8.68 and 7.06MPa, respectively. Both scaffold types exhibited mechanical properties within the range of human trabecular bone. However, the Gyroid scaffold demonstrated superior post-yield structural integrity.

Efstathiadis et al. [32] developed parametric CAD models of biomimetic Voronoi lattices inspired by sea urchin stereom. Node count, strut thickness, and geometric smoothness controlled the lattice geometry. The structures were fabricated by FFF using PLA, PLA/PHA, and wood-fiber-reinforced PLA/PHA, and evaluated through compression tests and finite element analysis. Although minor printing defects were identified and printed structures were visually compared with CAD models, geometric accuracy was not quantified, underscoring the necessity to assess both mechanical performance and manufacturing precision of stochastic porous structures.

Alemayehu and Todoh [33] developed composite TPMS gyroid lattices by combining conventional and walled gyroids through Boolean subtraction in nTop. PLA lattices with relative densities of 14% to 54% were fabricated by FFF and evaluated through compression tests, finite element analysis, and microscopy. Relative density significantly influenced deformation, stiffness, and energy absorption. In simulated automotive crash boxes, 25% relative density provided a balance between deformation capacity and rigidity, while 33%, 38%, and 54% showed potential for shock absorption.

Liu et al. [34] investigated the microstructure and compressive behavior of PLA/PHA–wood composite lattices fabricated by fused filament fabrication (FFF). X-ray microtomography revealed process-induced porosity and surface roughness, which caused local stress concentrations and reduced mechanical performance. Compression tests and finite element analysis showed that CAD-based models overestimated stiffness because they did not account for manufacturing defects. An increase in relative density partially compensated for the loss of mechanical performance. These findings highlight the importance of manufacturing defects and the need to assess differences between designed and fabricated porous structures.

Seto et al. [35] examined eleven porous structures derived from ten unit-cell geometries. Finite element analysis evaluated load-bearing capacity, while mass, surface area, production cost, time, and manufacturability were also assessed. The structures were fabricated using PLA with 100% infill and a layer thickness of 0.1mm. A titanium structure with 1mm wall and strut thicknesses was also fabricated. The results showed that unit-cell geometry influences mechanical performance and manufacturing efficiency, while wall and layer thicknesses are critical to fabrication accuracy.

Sharif Ullah [36] proposed a design-for-additive-manufacturing methodology for creating stochastic porous structures. Monte Carlo simulations were employed to generate point distributions across multiple layers. These distributions were subsequently converted into CAD models with randomly distributed pores through the application of concave-hull operations. Thin-walled and cascaded porous structures were fabricated utilizing commercial additive manufacturing (AM) equipment. Visual inspection indicated qualitative agreement between the fabricated structures and their corresponding CAD models.

Sharif Ullah et al. [37] introduced a self-contained system designed for the automated creation and additive manufacturing of stochastic porous structures. This system generated random point clouds within a defined boundary and directly produced STL datasets for fabrication. The resulting structures were manufactured using AM equipment and exhibited pores of random sizes and distributions, which permitted the passage of gases and liquids. The study further demonstrated that pore size could be modulated by adjusting the density of the point cloud, thereby highlighting potential applications in engineering and biomedical fields.

Sharif Ullah et al. [38] introduced a fractal-based method for the design of stochastic porous structures. Affine transformations within an iterated function system (IFS) generated point clouds exhibiting adjustable self-similarity and randomly sized and distributed pores. Voxel-based modeling was employed to convert the point clouds into three-dimensional CAD models. The approach was demonstrated using the Sierpiński carpet, and the resulting structures were fabricated through 3D printing. However, voxel-based conversion may produce disconnected or loose shells, which presents challenges for fabrication.

Sasaki and Ura [39] investigated geometric modeling of stochastic porous structures using triangular tessellation. Three methods were examined: tunneling, point cloud-based modeling [37], and a hybrid of the two. The tunneling method randomly removes triangles to control porosity from a plane, while the point cloud-based method generates irregular pores with variable sizes. The hybrid method combines both approaches to generate structures with irregular pore shapes and relatively consistent porosity. Slicing simulations showed qualitative agreement between the designed structures and their simulated fabrication results. It has been reported that both methods exhibit loose shells and thin walls in the porous structures. However, physical fabrication and quantitative evaluation of geometric accuracy were not conducted.

Kutari [40] proposed a C-K theory-based method for porous structure design using fractals. The method uses creative definitional knowledge, informal inductive knowledge, and deductive knowledge to derive algorithms that generate level-controlled point clouds of fractal shapes, including the Sierpinski pentagon, the Dürer’s pentagon, and the McWorter pentigree. Primitive polygons are created from the point clouds and arranged through scaling and rotation to prevent loose shells and thin walls. The arranged primitives are then converted into porous CAD models through geometric operations such as rotation, extraction, addition, and subtraction. However, the resulting structures exhibit deterministic pore clusters.

Table 1 provides a summary of previous studies focused on the design and fabrication of porous structures. However, the automated design, fused filament fabrication (FFF), and quantitative accuracy assessment of thin PLA structures with stochastic polygonal pores have not been sufficiently investigated. The remainder of this paper aims to address these research gaps.

## 3. Proposed Design Method

This section presents the proposed CAD method for creating stochastic porous structures, including the geometric definition of polygonal pores, their stochastic distribution within a planar cell array, the construction of three-dimensional models, and the analytical evaluation of porosity.

### 3.1. Creating a Pore

Pores are the fundamental building blocks of a porous structure. This study adopts a novel method to create individual pores, as described below.

**Figure 1:**
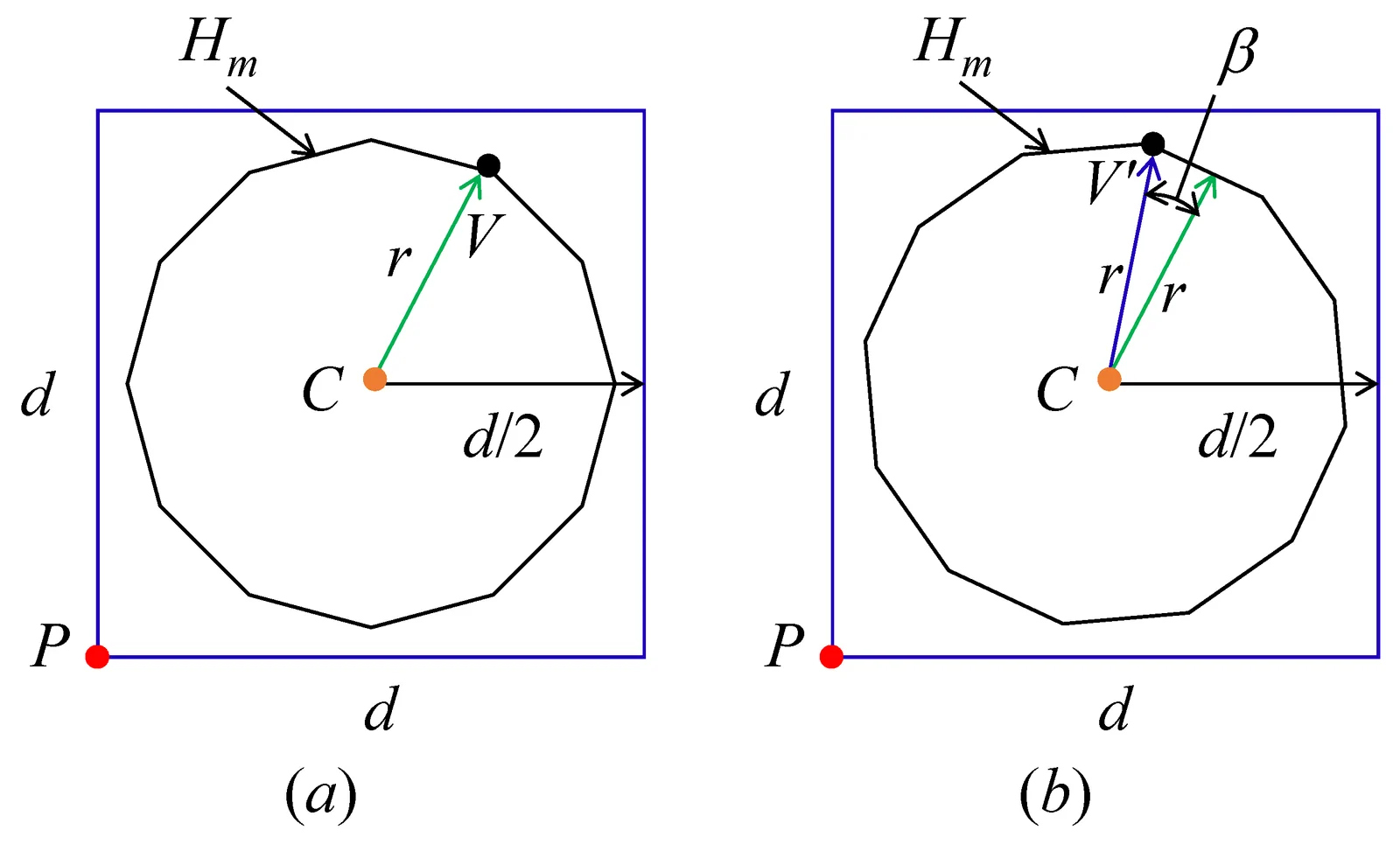
Schematic representation of the polygonal pore: (**a**) configuration without rotation and (**b**) configuration after rotation by an angle β.

The core concept for creating a pore is schematically illustrated in Figure 1. As shown in Figure 1, a polygonal pore is created on a planar square cell of dimensions d×d, where $d\in R_{>0}$ denotes the side length. Let the bottom-left corner of the cell be denoted by $P=\left(x,y\right)\in R^{2}$. The center of the cell is denoted by *C* and is defined as

(1) $$C=\left(x+\frac{d}{2},y+\frac{d}{2}\right)\in R^{2}.$$

Thus, *C* is located at a distance d/2 from each side of the square cell.

Within the cell, consider a regular polygonal pore denoted by $H_{m}$, whose center coincides with *C*. The integer-valued parameter *m* specifies the number of sides of the polygon and therefore determines the shape of the pore. It satisfies

(2) $$m\in Z_{\geq 3}.$$

For example, m=3 defines a triangular pore, m=4 defines a quadrilateral pore, m=5 defines a pentagonal pore, and likewise.

Let $V\in R^{2}$ denote a vertex of $H_{m}$. The distance from the center *C* to *V* is denoted by *r* and is defined as

(3) $$r=\alpha \frac{d}{2},\;0<\alpha <1,$$

where α∈(0,1)⊂R is a dimensionless size parameter. Consequently,

(4) $$0<r<\frac{d}{2}.$$

For a regular polygon with *m* sides, the angular positions of its vertices are defined by

(5) $$\theta \in \left\{\frac{2\pi k}{m}\;|\;k=0,1,\ldots ,m-1\right\},$$

where θ∈[0,2π) is measured in radians. Accordingly, the position of an arbitrary vertex *V* is expressed as

(6) $$V=C+r\left[\begin{array}{c} \cos\theta \\ \sin\theta \end{array}\right].$$

The polygonal pore $H_{m}$ can be rotated counterclockwise about *C* through an angle β. The rotation angle β is a real-valued parameter bounded by

(7) $$\beta \in \left[\beta_{\min},\beta_{\max}\right],\;0\leq \beta_{\min}<\beta_{\max}\leq 2\pi ,\;\beta_{\min},\beta_{\max}\in R.$$

After rotation, the vertex *V* moves to $V^{\prime }\in R^{2}$, whose position is given by

(8) $$V^{\prime }=C+r\left[\begin{array}{c} \cos(\theta +\beta )\\ \sin(\theta +\beta ) \end{array}\right].$$

Therefore, the geometry of the pore $H_{m}$ is controlled by the three parameters

(9) m,α,β,

where $m\in Z_{\geq 3}$ controls the pore shape, α∈(0,1) controls the relative pore size, and $\beta \in [\beta_{\min},\beta_{\max}]$, where $0\leq \beta_{\min}<\beta_{\max}\leq 2\pi$, controls the pore orientation. Stochastic pore geometries can therefore be generated by assigning these parameters according to prescribed random distributions.

However, the formulation can also be extended from a square cell to a rectangular cell of dimensions $d_{1}\times d_{2}$, where $d_{1},d_{2}\in R_{>0}$. In this case, the pore center is located at the center of the rectangular cell, and the circumradius must satisfy $0<r<\frac{1}{2}\min\left(d_{1},d_{2}\right)$ so that the polygonal pore remains within the cell boundary. Furthermore, instead of prescribing arbitrary lower and upper bounds $\beta_{\min}$ and $\beta_{\max}$, the rotation angle may be selected from the nonredundant interval β∈[0,2π/m), which follows from the rotational symmetry of a regular polygon with *m* sides.

### 3.2. Stochastic Porous Structure

Based on the pore formulation described in the previous subsection, a stochastic porous structure design method is developed as follows.

A stochastic porous structure is generated by subdividing a planar domain into square cells of dimensions d×d. A polygonal pore $H_{m(i,j)}$ is defined within each cell (i,j). The pore shape, size, and orientation are controlled by m(i,j), $\alpha_{ij}$, and $\beta_{ij}$, respectively. The polygonal pore is extruded through the thickness *c* to form a three-dimensional void. The side length of each square cell is denoted by $d\in R_{>0}$. The numbers of cells in the *x*- and *y*-directions are denoted by $n_{x},n_{y}\in Z_{>0}$. The thickness of the solid is denoted by $c\in R_{>0}$, and the origin of the planar domain is defined by $\left(x_{0},y_{0}\right)\in R^{2}$. The cell indices are given by $i\in \{0,1,\ldots ,n_{x}-1\}$ and $j\in \{0,1,\ldots ,n_{y}-1\}$. The cell coordinates are

(10) $$P_{ij}=\left(x_{i}=x_{0}+id,y_{j}=y_{0}+jd\right).$$

The cell center is

(11) $$C_{ij}=\left(x_{i}+\frac{d}{2},y_{j}+\frac{d}{2}\right).$$

The polygon parameter $m_{ij}$ is an integer that determines the number of sides of the pore and is bounded by

(12) $$m_{ij}\in \left\{m_{\min},m_{\min}+1,\ldots ,m_{\max}\right\},\;m_{\min},m_{\max}\in Z_{\geq 3}.$$

The pore-size parameter $\alpha_{ij}$ is a real-valued parameter bounded by

(13) $$\alpha_{ij}\in \left[\alpha_{\min},\alpha_{\max}\right],\;0<\alpha_{\min}<\alpha_{\max}<1,\;\alpha_{\min},\alpha_{\max}\in R.$$

The corresponding vertex radius $r_{ij}$ is defined by

(14) $$r_{ij}=\alpha_{ij}\frac{d}{2},\;r_{ij}\in \left[\alpha_{\min}\frac{d}{2},\alpha_{\max}\frac{d}{2}\right].$$

For a polygon with $m_{ij}$ sides, the vertex index is defined by $k\in \{0,1,\ldots ,m_{ij}-1\}$.

The angular position of the *k*-th vertex is given by

(15) $$\theta_{k}=\frac{2\pi k}{m_{ij}},\;\theta_{k}\in \left[0,2\pi \right),$$

where $\theta_{k}$ is measured in radians.

The position of the unrotated *k*-th vertex is given by

(16) $$V_{k}\left(i,j\right)=C_{ij}+r_{ij}\left[\begin{array}{c} \cos\theta_{k}\\ \sin\theta_{k} \end{array}\right].$$

The counterclockwise rotation angle $\beta_{ij}$ is a real-valued parameter bounded by

(17) $$\beta_{ij}\in \left[\beta_{\min},\beta_{\max}\right],\;0\leq \beta_{\min}<\beta_{\max}\leq 2\pi ,\;\beta_{\min},\beta_{\max}\in R.$$

Accordingly, the position of the rotated *k*-th vertex is given by

(18) $$V_{k}^{\prime }\left(i,j\right)=C_{ij}+r_{ij}\left[\begin{array}{c} \cos(\theta_{k}+\beta_{ij})\\ \sin(\theta_{k}+\beta_{ij}) \end{array}\right].$$

The pore-generation probability *p* is a real-valued parameter with p∈(0,1]. For each cell, a random number *q* is sampled from a uniform distribution, q∼U(0,1). A pore is generated if q<p.

**Figure 2:**
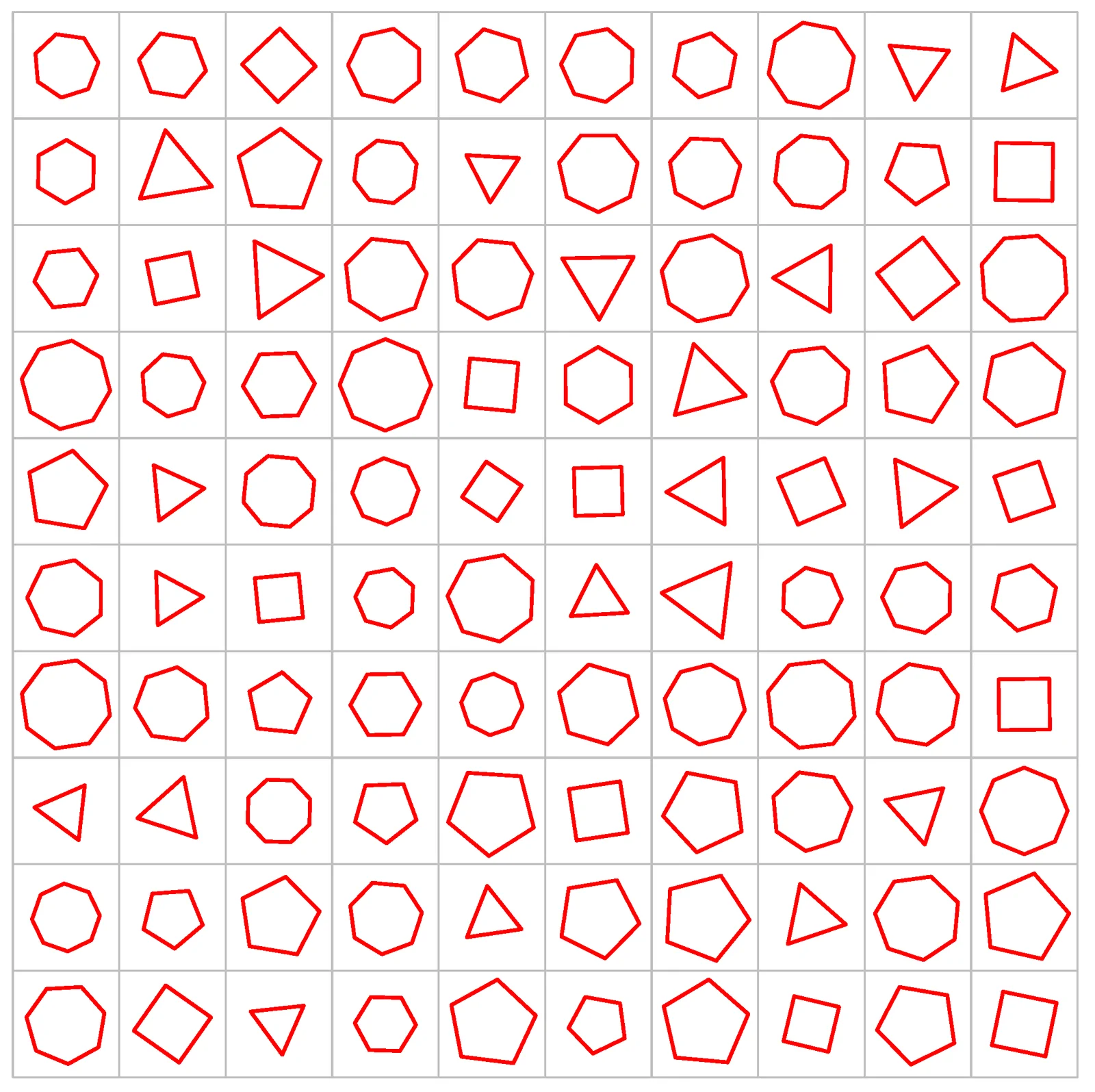
Discretized grid on the slab surface with different types of polygonal pores generated using stochastic pore-shape, pore-size, and orientation parameters. In this example, p=1, m∈{3,4,…,8}, α∈[0.56,0.86), and β∈[0,2π).

For example, when the pore-generation probability is set to p=1, a pore is generated in every cell. In the illustrative case shown in Figure 2, the polygon parameter is randomly selected as m∈{3,4,…,8}, the pore-size parameter is randomly selected in the range α∈[0.56,0.86), and the rotation angle is randomly selected in the range β∈[0,2π). The resulting porous structure model is shown in Figure 2.

**Figure 3:**
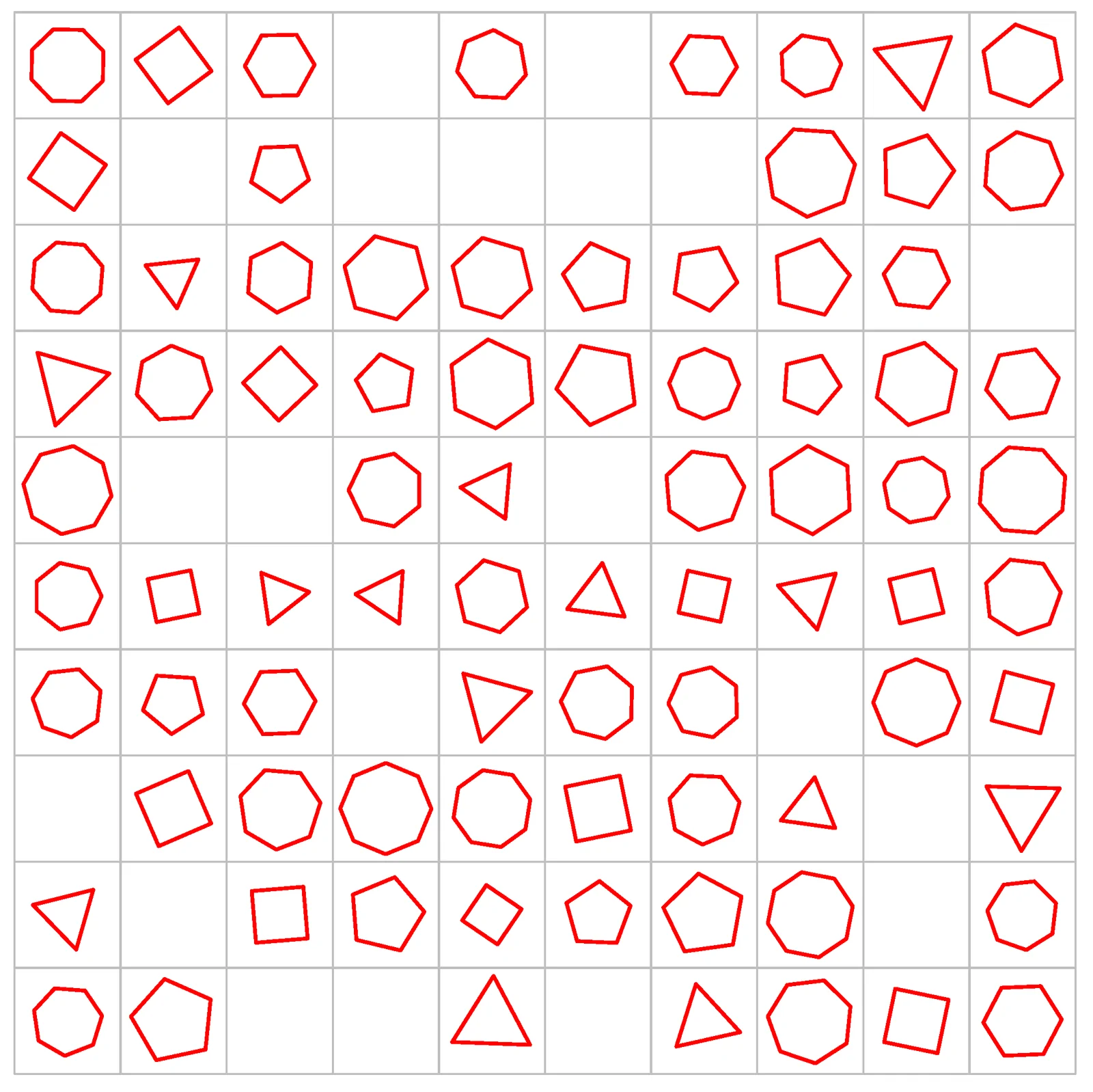
Discretized grid on the slab surface with polygonal pores generated using stochastic pore-shape, pore-size, and orientation parameters. In this example, p=0.8, m∈{3,4,…,8}, α∈[0.56,0.86), and β∈[0,2π).

On the other hand, Figure 3 shows a model of a stochastic porous structure generated with p=0.8, while the pore shape, size, and orientation are randomly varied within the prescribed range corresponding to Figure 2.

Algorithm 1 outlines the procedure for generating the three-dimensional stochastic porous structure and its corresponding STL data. First, a rectangular solid is created using the prescribed cell size, number of cells, and thickness. Each cell is then evaluated using the pore-generation probability *p*. If a pore is assigned to a cell, the polygon type $m_{ij}$, size parameter $\alpha_{ij}$, and rotation angle $\beta_{ij}$ are selected within the prescribed ranges. The vertex coordinates of the polygonal pore are then computed, and the polygon is extruded through the solid’s thickness to form a three-dimensional void. This void is subtracted from the rectangular solid via a Boolean difference operation. The procedure is repeated for all cells to obtain the final stochastic porous structure. The algorithm can be implemented in a script-based CAD system to automatically generate the required porous structure. In this study, OpenSCAD, a script-based CAD system, is used to implement the algorithm and export the resulting stochastic porous structure as STL data for subsequent 3D printing. The complete OpenSCAD source code used in this study is provided in Supplementary Material S1.

**Figure 4:**
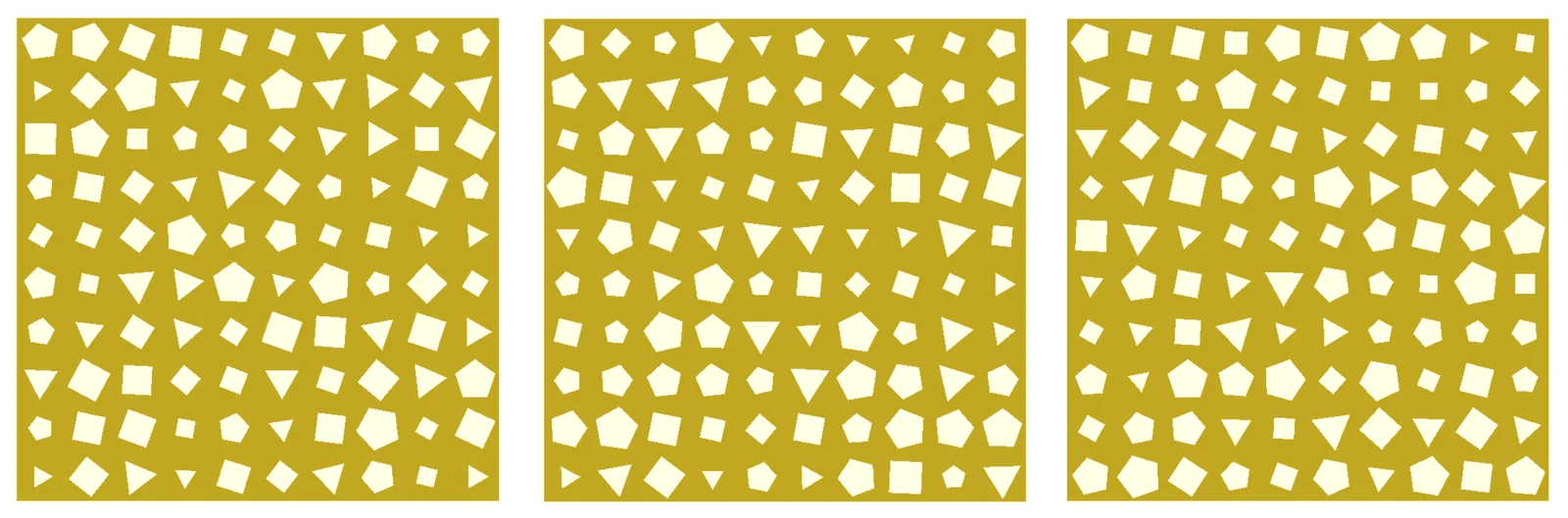
Three CAD models of stochastic porous structures generated using Algorithm 1, together with their corresponding PLA-based 3D-printed structures. The same design conditions were used for all models: d=5mm, $n_{x}=n_{y}=10$, c=1mm, and p=1. The stochastic parameters were specified as $m_{ij}\in \left\{3,4,5\right\}$, $\alpha_{ij}\in \left[0.50,0.95\right]$, and $\beta_{ij}\in \left[0,\pi /4\right]$. The differences among the models arise from the stochastic selection of pore shape, size, and orientation in the individual cells. The corresponding PLA-based 3D-printed structures are shown beneath the CAD models.

The three CAD models of stochastic porous structures shown in Figure 4 were obtained by running the code based on Algorithm 1 three times, changing the seed for random number generation. The 3D-printed counterparts are also shown in Figure 4. The same design conditions were used for all three models: d=5mm, $n_{x}=n_{y}=10$, c=1mm, and p=1, so that a pore was generated in every cell. The stochastic pore parameters were specified as $m_{ij}\in \left\{3,4,5\right\}$, $\alpha_{ij}\in \left[0.50,0.95\right]$, and $\beta_{ij}\in \left[0,\pi /4\right]$. Although the prescribed parameter ranges were identical, the resulting CAD models differ because the pore shape, size, and orientation were selected randomly for each cell. These results show that Algorithm 1 can generate geometrically different porous structures under the same design conditions. The generated CAD models were subsequently used to fabricate PLA-based 3D-printed porous structures. The printing conditions and manufacturing specifications are presented in the following section. Thus, the present section focuses on the CAD-based generation of stochastic porous geometries, while the manufacturing characteristics and fabrication results are discussed separately.
**Algorithm 1** Generation of a Stochastic Porous Structure  1:**Input:** $d\in R_{>0}$, $n_{x},n_{y}\in Z_{>0}$, $c\in R_{>0}$, $m_{\min},m_{\max}\in Z_{\geq 3}$, $\alpha_{\min},\alpha_{\max}\in \left(0,1\right)$, p∈(0,1], and $\left(x_{0},y_{0}\right)\in R^{2}$  2:**Output:** Stochastic porous solid *B*  3:Initialize *B* as a rectangular solid of dimensions $n_{x}d\times n_{y}d\times c$ with origin $\left(x_{0},y_{0},0\right)$  4:**for** i=0 to $n_{x}-1$ **do**  5:      **for** j=0 to $n_{y}-1$ **do**  6:         Generate q∼U(0,1)  7:         **if** q<p **then**  8:            $x_{i}\leftarrow x_{0}+id$  9:            $y_{j}\leftarrow y_{0}+jd$10:            $P_{ij}\leftarrow \left(x_{i},y_{j}\right)$11:            $C_{ij}\leftarrow \left(x_{i}+\frac{d}{2},y_{j}+\frac{d}{2}\right)$12:            Randomly select $m_{ij}\in \left\{m_{\min},m_{\min}+1,\ldots ,m_{\max}\right\}$13:            Randomly select $\alpha_{ij}\in \left[\alpha_{\min},\alpha_{\max}\right]$14:            $r_{ij}\leftarrow \alpha_{ij}\frac{d}{2}$15:            Randomly select $\beta_{ij}\in \left[0,2\pi \right)$16:            **for** k=0 to $m_{ij}-1$ **do**17:                $\theta_{k}\leftarrow \frac{2\pi k}{m_{ij}}$18:                $V_{k}^{\prime }\left(i,j\right)\leftarrow C_{ij}+r_{ij}\left[\begin{array}{c} \cos\left(\theta_{k}+\beta_{ij}\right)\\ \sin\left(\theta_{k}+\beta_{ij}\right) \end{array}\right]$19:            **end for**20:            Construct the polygonal pore $H_{m_{ij}}$ from the vertices $V_{k}^{\prime }\left(i,j\right)$21:            Extrude $H_{m_{ij}}$ through the thickness *c* to obtain the three-dimensional void $V_{ij}$22:            $B\leftarrow B\setminus V_{ij}$23:         **end if**24:      **end for**25:**end for**26:**return** *B*

### 3.3. Porosity of a Model

The porosity of each stochastic porous structure is calculated from the total volume of the polygonal pores relative to the volume of the initial nonporous plate. Since each pore is defined as a regular polygon and is extruded through the full plate thickness *c*, its volume can be obtained analytically from its cross-sectional area.

For a pore located in cell (i,j), the regular polygon has $m_{ij}$ sides and a circumradius of $r_{ij}$. Its cross-sectional area is given by

(19) $$A_{ij}=\frac{m_{ij}}{2}r_{ij}^{2}\sin\left(\frac{2\pi }{m_{ij}}\right).$$

Since the polygonal pore is extruded through the plate thickness *c*, the corresponding pore volume is

(20) $$V_{ij}=A_{ij}c=\frac{m_{ij}}{2}r_{ij}^{2}\sin\left(\frac{2\pi }{m_{ij}}\right)c.$$

From the pore-generation formulation, $r_{ij}=\alpha_{ij}d/2$. Substituting this relation into Equation (20) gives

(21) $$V_{ij}=\frac{m_{ij}\alpha_{ij}^{2}d^{2}c}{8}\sin\left(\frac{2\pi }{m_{ij}}\right).$$

The total volume of all generated pores is obtained by summing the individual pore volumes over all cells. Let $\delta_{ij}$ denote the pore-generation state of cell (i,j), where $\delta_{ij}=1$ indicates that a pore is generated and $\delta_{ij}=0$ indicates otherwise. The total pore volume is therefore

(22) $$V_{\mathrm{pore}}=\sum_{i=0}^{n_{x}-1}\sum_{j=0}^{n_{y}-1}\delta_{ij}V_{ij}.$$

For p=1, a pore is generated in every cell and hence $\delta_{ij}=1$ for all *i* and *j*.

The volume of the initial nonporous plate is

(23) $$V_{0}=n_{x}n_{y}d^{2}c.$$

Accordingly, the porosity ϕ is calculated as

(24) $$\phi =\frac{V_{\mathrm{pore}}}{V_{0}}\times 100\%.$$

By substituting the expressions for the pore volume and the initial volume, the porosity can be expressed as follows:

(25) $$\phi =\frac{\sum_{i=0}^{n_{x}-1}\sum_{j=0}^{n_{y}-1}\delta_{ij}\frac{m_{ij}\alpha_{ij}^{2}d^{2}c}{8}\sin\left(\frac{2\pi }{m_{ij}}\right)}{n_{x}n_{y}d^{2}c}\times 100\%.$$

Since $d^{2}c$ is a common factor in both the numerator and denominator, the porosity expression can be simplified as follows:

(26) $$\phi =\frac{1}{8n_{x}n_{y}}\sum_{i=0}^{n_{x}-1}\sum_{j=0}^{n_{y}-1}\delta_{ij}m_{ij}\alpha_{ij}^{2}\sin\left(\frac{2\pi }{m_{ij}}\right)\times 100\%.$$

Equation (26) shows that the resulting porosity is directly influenced by the stochastic pore-shape parameter $m_{ij}$, the stochastic pore-size parameter $\alpha_{ij}$, and the pore-generation state $\delta_{ij}$. In contrast, the rotation parameter $\beta_{ij}$ changes only the orientation of the polygonal pore and does not change its area or volume. Therefore, the stochastic variation in the pore-generation parameters produces corresponding stochastic variation in the porosity of the generated structures.

**Figure 5:**
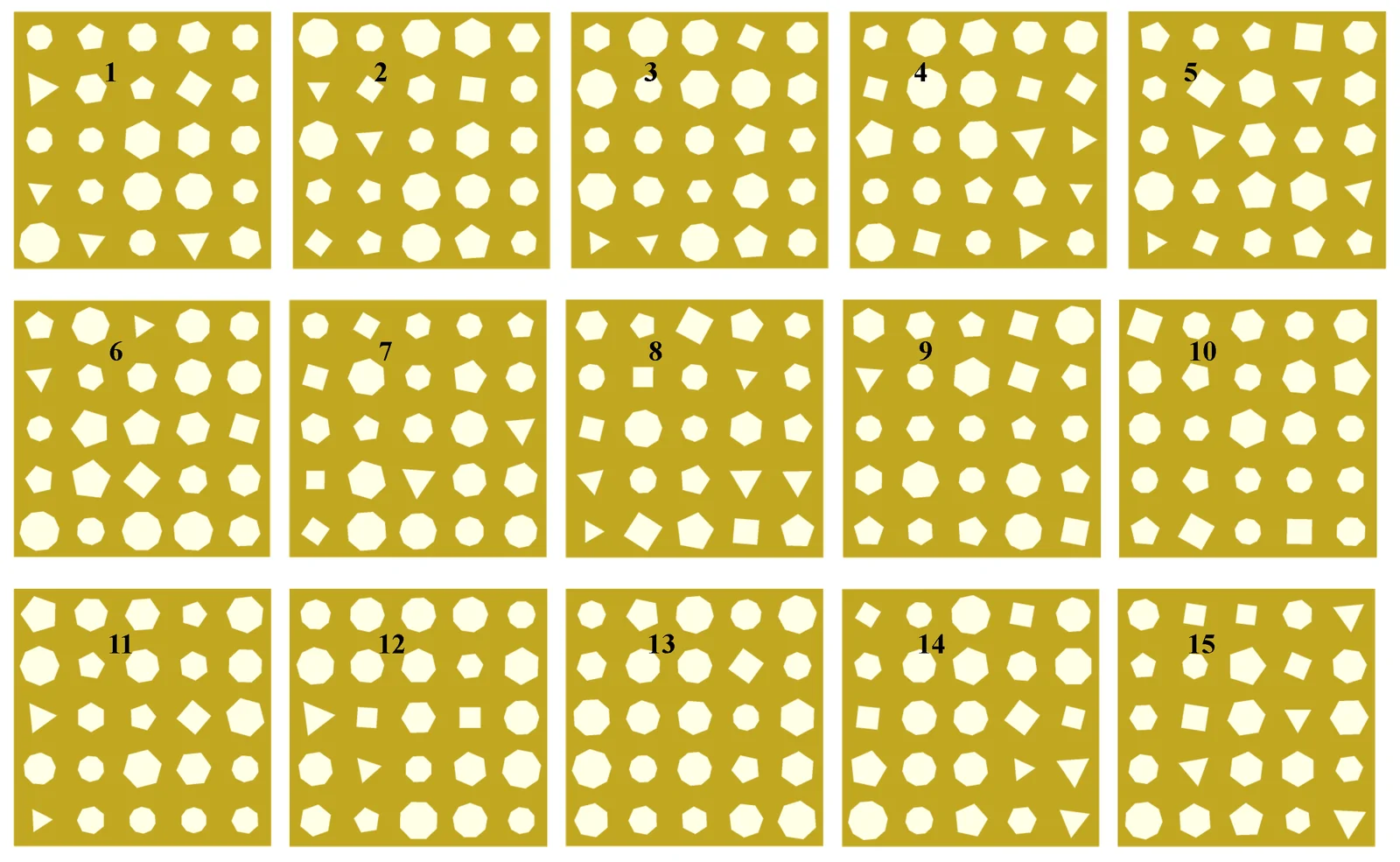
Fifteen CAD models of stochastic porous structures generated using Algorithm 1. The same design conditions were used for all models: d=4mm, $n_{x}=n_{y}=5$, c=0.2mm, and p=1. The stochastic parameters were specified as $m_{ij}\in \left\{3,4,\ldots ,10\right\}$, $\alpha_{ij}\in \left[0.50,0.80\right]$, and $\beta_{ij}\in \left[0,\pi /4\right]$. The differences among the models arise from the stochastic selection of pore shape, size, and orientation in the individual cells.

Figure 5 shows fifteen CAD models of stochastic porous structures generated using Algorithm 1. The same design conditions were used for all models: d=4mm, $n_{x}=n_{y}=5$, c=0.2mm, and p=1. The stochastic parameters were specified as $m_{ij}\in \left\{3,4,\ldots ,10\right\}$, $\alpha_{ij}\in \left[0.50,0.80\right]$, and $\beta_{ij}\in \left[0,\pi /4\right]$.

**Figure 6:**
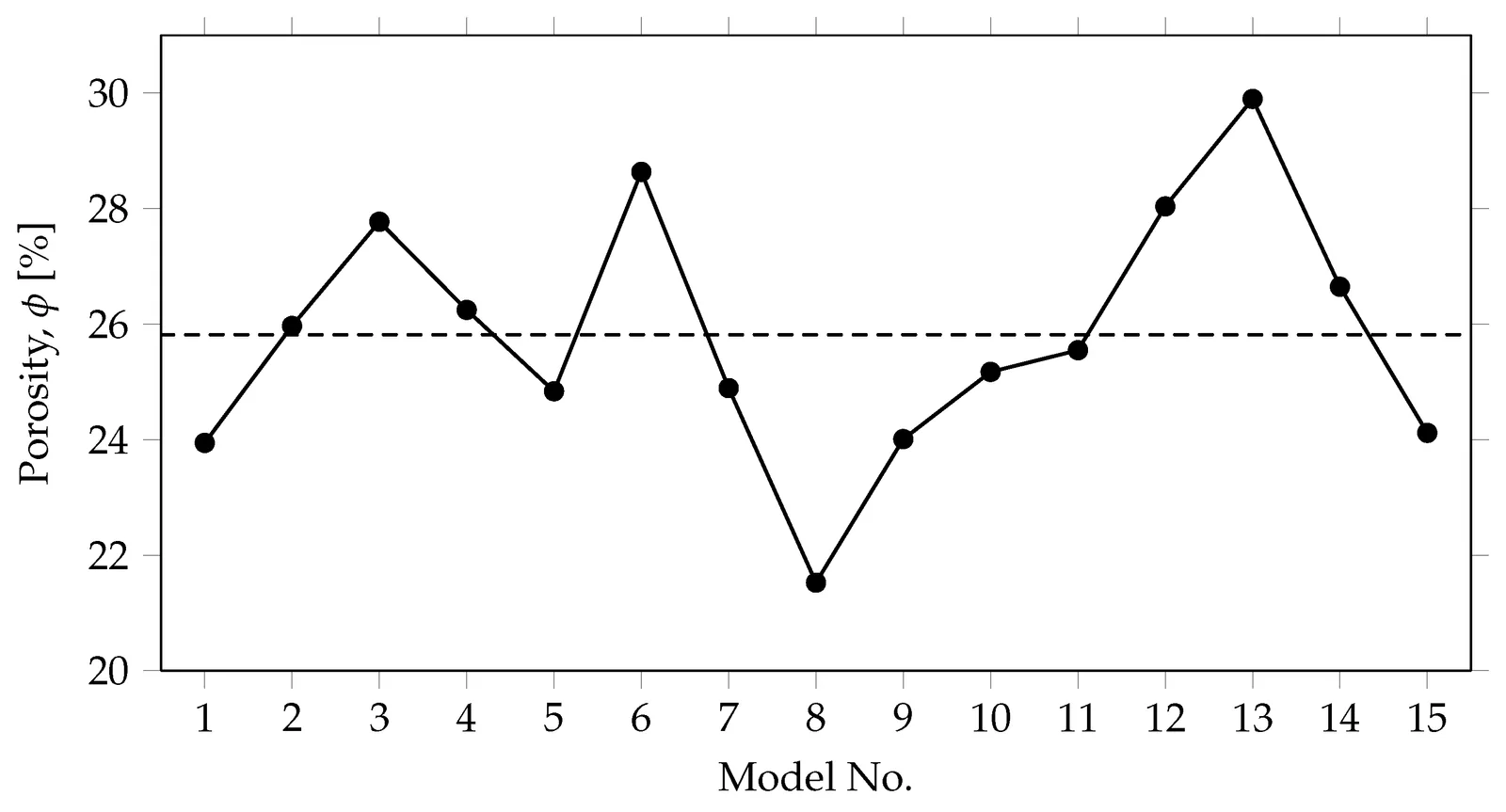
Porosity of the fifteen stochastic porous structure models. The dashed line indicates the average porosity of approximately 25.82%.

Although the same parameter ranges were used for all models, the resulting structures differ because the pore shape, size, and orientation were selected stochastically for the individual cells. Figure 6 shows the porosity of the fifteen stochastic porous structures presented in Figure 5. As shown in Figure 6, the porosity varies among the models, with an average porosity of approximately 25.82%.

However, a sensitivity analysis based on Equation (26) was performed to examine the influence of the stochastic pore parameters on the resulting porosity. The same parameter ranges used for the generated models were considered, namely $m_{ij}\in \left\{3,4,\ldots ,10\right\}$ and $\alpha_{ij}\in \left[0.50,0.80\right]$. For p=1, the pore-size parameter $\alpha_{ij}$ accounted for approximately 58.44% of the variation in porosity, whereas the pore-shape parameter $m_{ij}$ accounted for approximately 38.88%. Their interaction accounted for approximately 2.67%. The rotation angle $\beta_{ij}$ had no influence on porosity because it changes only the orientation of a pore without changing its area or volume. Therefore, among the geometric stochastic parameters, $\alpha_{ij}$ is the most influential parameter. In addition, the pore-generation probability *p* directly controls the expected porosity. For the present parameter ranges, the expected porosity is approximately E[ϕ]=26.433p%, demonstrating an approximately linear dependence on *p*.

An important interpretation of Equation (26) is that the cell size *d* and the plate thickness *c* are canceled during the simplification and therefore do not directly affect the porosity. Moreover, the rotation angle $\beta_{ij}$ does not appear in the final porosity expression because it changes only the orientation of the pore and not its area or volume. Consequently, the principal geometric parameters that control porosity are the pore-size parameter $\alpha_{ij}$ and the polygon parameter $m_{ij}$. Among these, $\alpha_{ij}$ has a stronger influence because it appears in Equation (26) as $\alpha_{ij}^{2}$.

## 4. Printability Results

This section presents the fabrication and accuracy evaluation of thin stochastic porous structures. It describes the printing conditions and experimental equipment, examines the effects of layer thickness and deposition passes on printability, and quantitatively evaluates the geometric agreement between the designed and fabricated structures using binary image comparison.

### 4.1. Equipment and Materials

The thin stochastic porous structures were fabricated using a commercially available 3D printer. The specifications of the printer are summarized in Table 2. As listed in Table 2, a Creality K2 Plus printer based on fused filament fabrication (FFF) was used with polylactic acid (PLA) filament. The printer was equipped with a 0.4mm nozzle and used 1.75mm-diameter filament. Layer thicknesses of 0.1mm and 0.2mm were considered. The infill rate was set to 100%, and no support structures were applied. Creality Print 6.0 was used for slicing and generating the material-deposition paths.

The fabricated stochastic porous structures were observed using a digital microscope. The specifications of the microscope are summarized in Table 3. As listed in Table 3, a KEYENCE VHX-5000 digital microscope equipped with a VH-ZST lens was used. The microscope provides magnifications from 20× to 2000× and uses a 1/1.8-inch CMOS image sensor with 1600×1200 effective pixels. The working distance was 15mm, and a high-intensity LED light source was used for illumination. The acquired images were stored in JPEG or TIFF format. In this study, the magnification was fixed at 20× for consistent observation of the fabricated structures.

### 4.2. Fabrication Results

**Figure 7:**
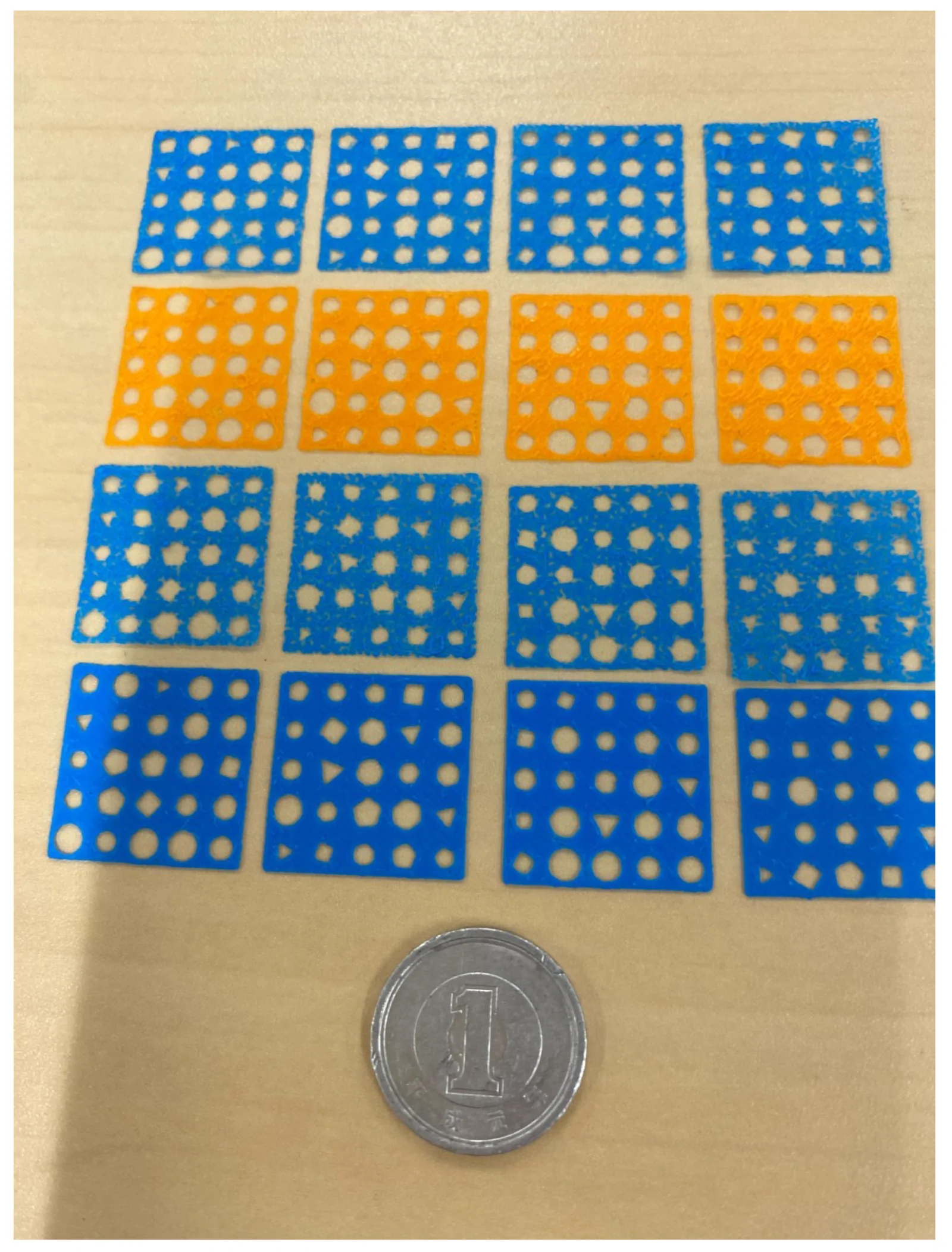
Photographs of the fabricated PLA specimens.

Four fabrication configurations were examined using a total of sixteen PLA specimens. For each configuration, four stochastic porous CAD models were prepared, and the corresponding material-deposition paths were generated using the slicing software specified in Table 2. The porous structures were then fabricated according to these deposition paths. Figure 7 shows photographs of the sixteen fabricated specimens arranged in four rows, with each row containing four specimens. The pictures of the porous structures from top to bottom, correspond to the first, second, third, and fourth (last) configurations, respectively. For all specimens, the fabrication was performed with a 0.4mm nozzle and an extrusion line width of 0.42mm. The infill density was 100%, the printing speed was 100mm/s, the nozzle temperature was 220 °C, and the build-plate temperature was 50 °C. These conditions were maintained throughout the experiments.

**Figure 8:**
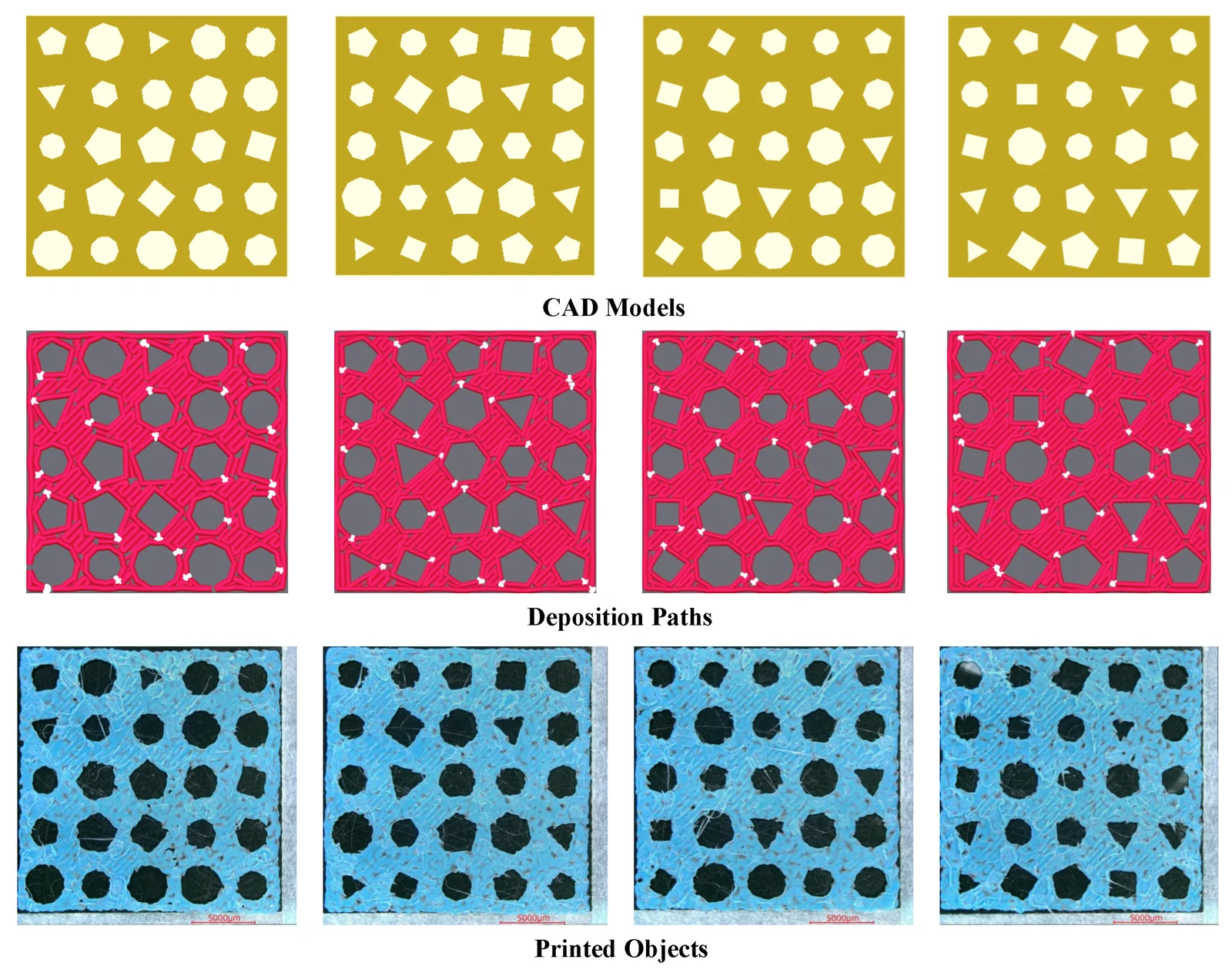
CAD models, material-deposition paths, and corresponding microscope images of the stochastic porous structures after completing a single deposition pass. The upper row shows the CAD models, the middle row shows the material-deposition paths generated by the slicing software, and the lower row shows microscope images of the fabricated specimens acquired at 20× magnification. The designed structure height was c=100μm, with a specified layer height of 100μm. Since the specimens were collected after completing the single deposition pass, their expected height was 100μm.

**Figure 9:**
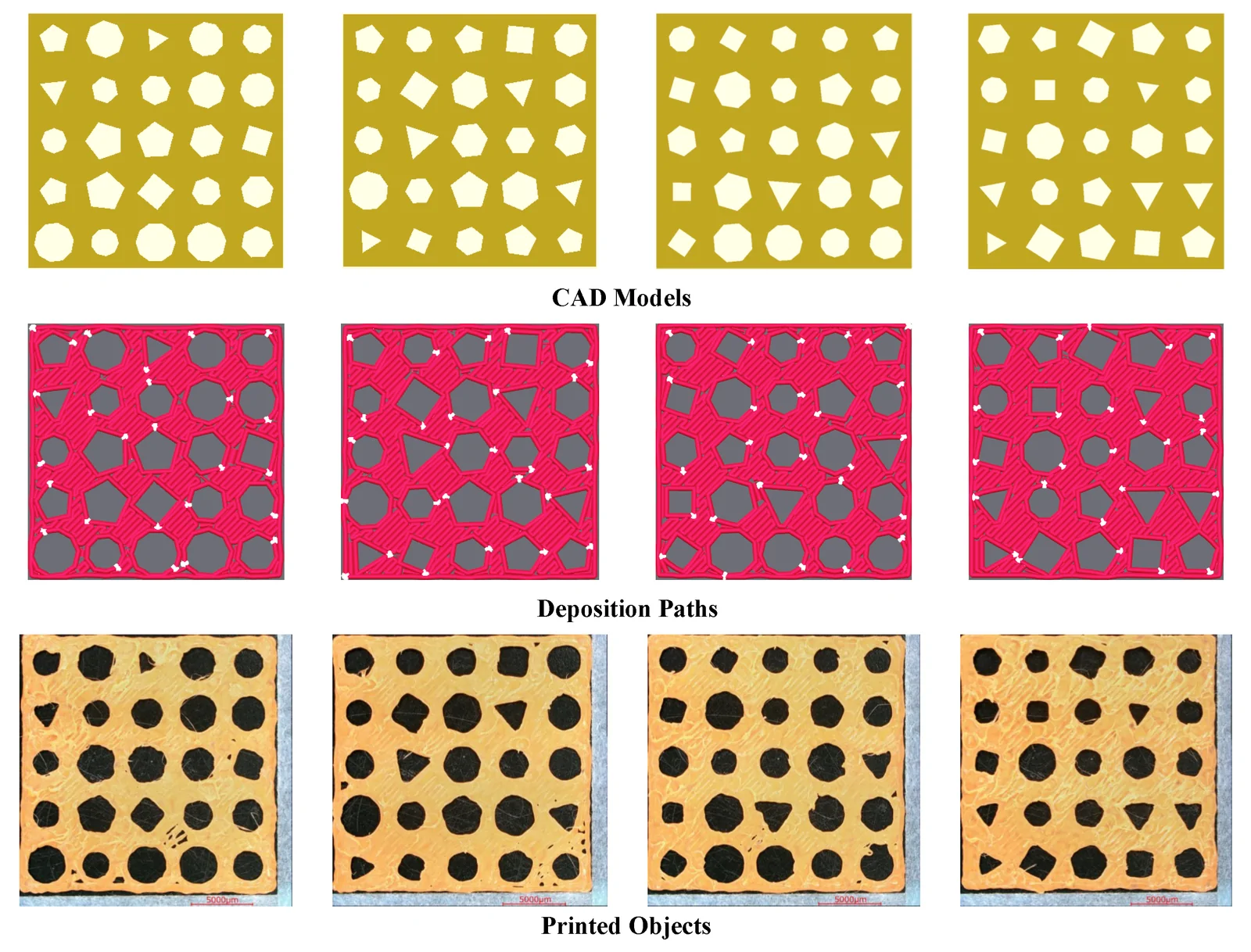
CAD models, material-deposition paths, and corresponding microscope images of the stochastic porous structures after completing a single deposition pass. The upper row shows the CAD models, the middle row shows the material-deposition paths generated by the slicing software, and the lower row shows microscope images of the fabricated specimens acquired at 20× magnification. The designed structure height was c=200μm, with a specified layer height of 200μm. Since the specimens were collected after completing the single deposition pass, their expected height was 200μm.

**Figure 10:**
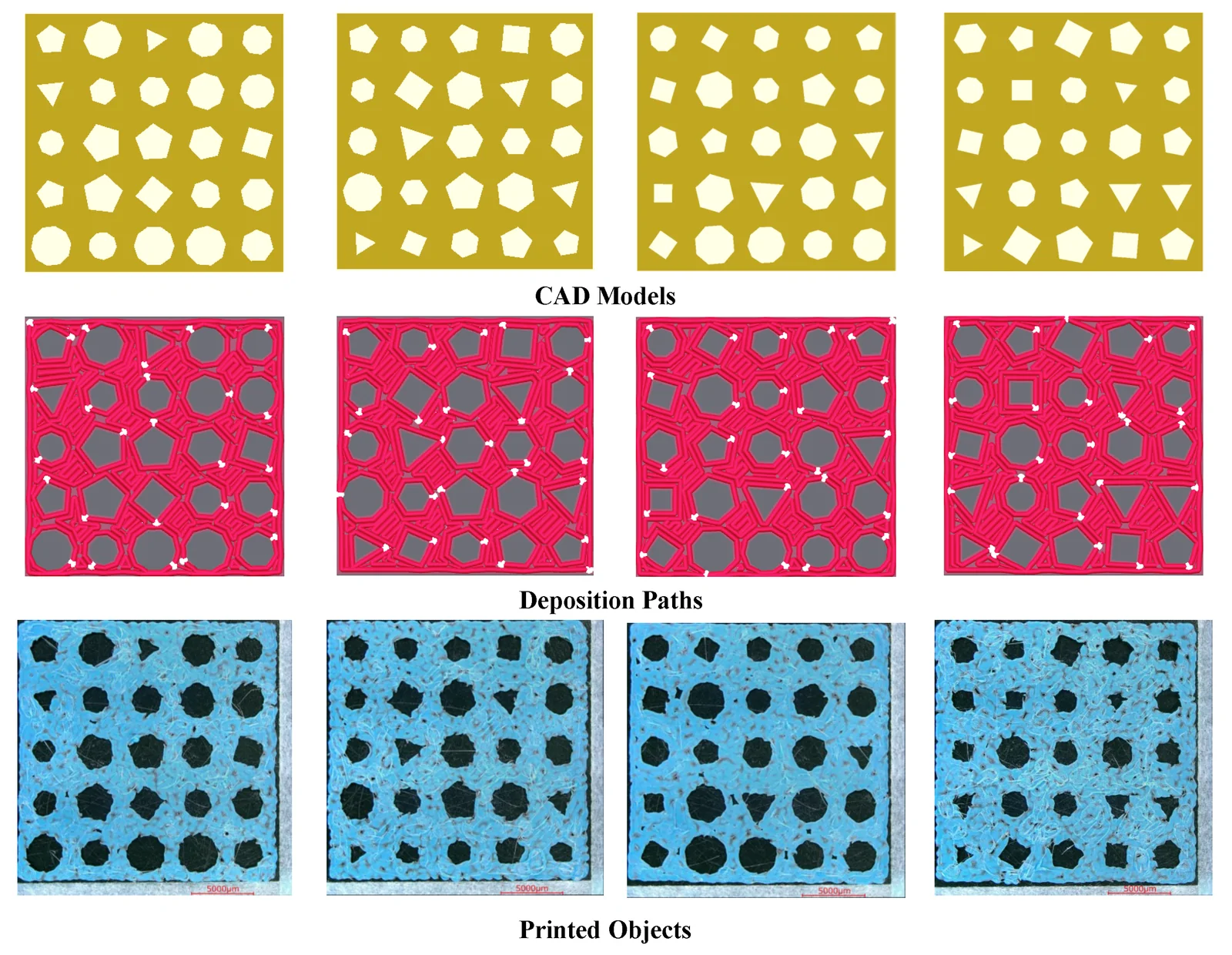
CAD models, material-deposition paths, and corresponding microscope images of the stochastic porous structures after the first deposition pass of the two-pass fabrication process. The designed structure height was c=200μm, with a specified layer height of 100μm. Since the specimens were collected after completing the first deposition pass, their expected height was 100μm.

**Figure 11:**
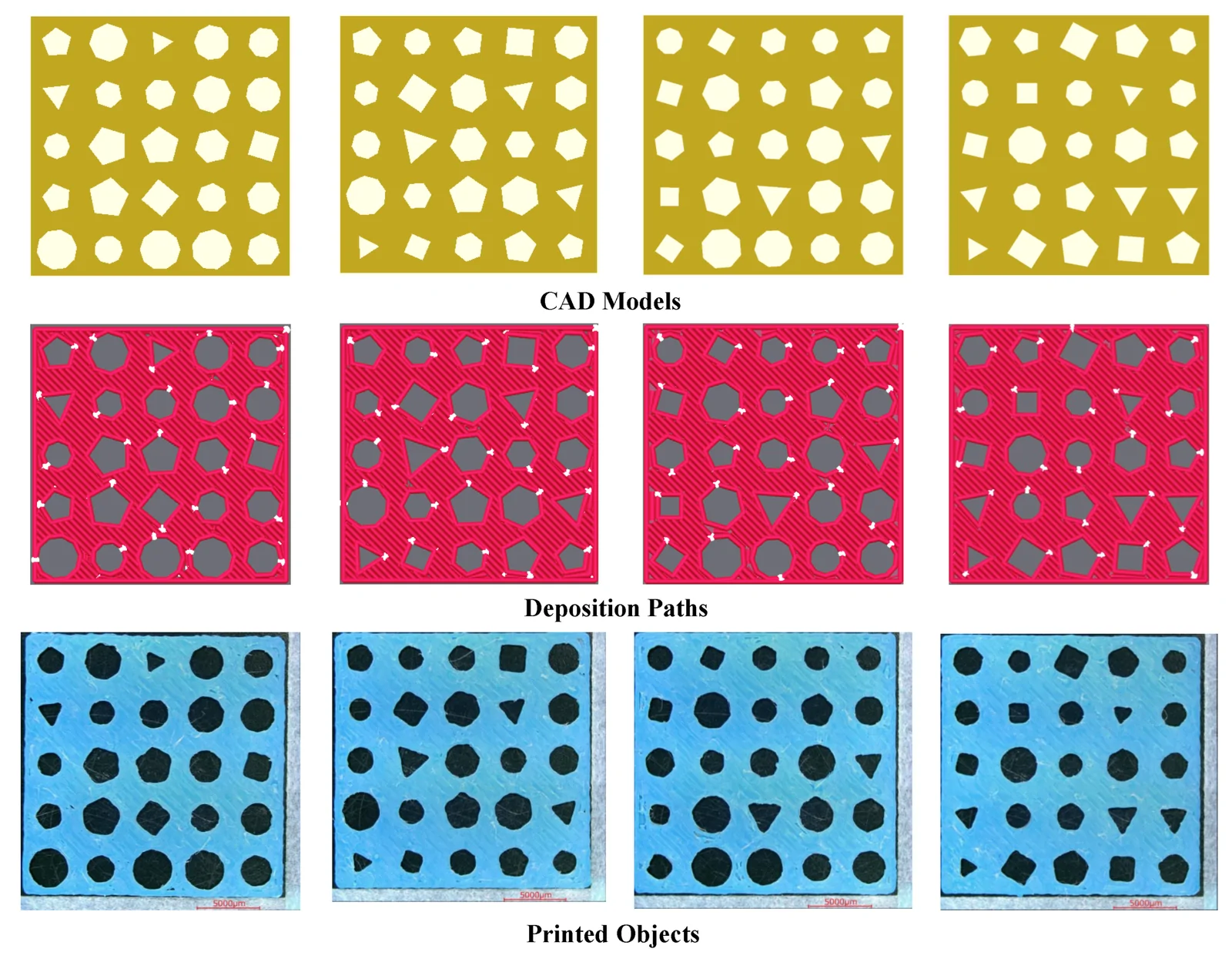
CAD models, material-deposition paths, and corresponding microscope images of the stochastic porous structures after the second deposition pass of the two-pass fabrication process. The designed structure height was c=200μm, with a specified layer height of 100μm. Since the specimens were collected after completing the second deposition pass, their expected height was 200μm.

The same geometric parameters were used for all four fabrication configurations: $a=n_{x}=n_{y}=20\;\mathrm{mm}$, n=5, and d=4mm. The stochastic parameters were specified as p=1, $m_{ij}\in \left\{3,4,\ldots ,10\right\}$, $\alpha_{ij}\in \left[0.50,0.80\right]$, and $\beta_{ij}\in \left[0,\pi /4\right]$. Setting p=1 ensured that a pore was generated in every cell. The designed structure thickness was 100μm for Figure 8 and 200μm for Figure 9, Figure 10 and Figure 11. Different stochastic realizations can be generated by changing the base random seed (see Supplementary Material S1).

The expected thickness of each specimen is described as follows. For Figure 8, the model thickness and specified layer thickness were both 100μm, requiring a single deposition pass to complete the fabrication. The specimens were collected after completing this single pass. Thus, their expected thickness was 100μm. Similarly, for Figure 9, the model thickness and specified layer thickness were both 200μm, requiring a single deposition pass to complete the fabrication. The specimens were collected after completing this pass. Thus, their expected thickness was 200μm. In contrast, the specimens shown in Figure 10 and Figure 11 were designed with a model thickness of 200μm and a specified layer thickness of 100μm. Therefore, two deposition passes were required to complete the fabrication. The specimens shown in Figure 10 were collected after completing the first pass, before depositing the second layer. Thus, their expected thickness was 100μm, although the model thickness was 200μm. On the other hand, the specimens shown in Figure 11 were collected after completing both deposition passes, resulting in an expected final thickness of 200μm.

Figure 8 shows the results of the first configuration, including the CAD models (top), material-deposition paths (middle), and microscope images of the fabricated specimens (bottom) for the four specimens, numbered 1–4 from left to right. In this fabrication configuration, the designed structure height was c=100μm, with a specified layer height of 100μm. Therefore, a single deposition pass was required to complete the fabrication. The specimens were collected after completing this deposition pass, and thus their expected height was 100μm. The filament consumption was approximately 0.09g per specimen, while the printing times for Specimens 1–4 were 89, 92, 85, and 86s, respectively.

Figure 9 shows the results of the second configuration, including the CAD models (top), material-deposition paths (middle), and microscope images of the fabricated specimens (bottom) for the four specimens, numbered 1–4 from left to right. In this fabrication configuration, the designed structure height was c=200μm, with a specified layer height of 200μm. Therefore, a single deposition pass was required to complete the fabrication. The specimens were collected after completing this deposition pass, and thus their expected height was 200μm. The filament consumption was approximately 0.12g per specimen, while the printing times for Specimens 1–4 were 90, 88, 85, and 91s, respectively.

Figure 10 presents the results of the third configuration, displaying the CAD models, material-deposition paths, and microscope images of the four fabricated specimens, numbered 1 to 4 from left to right. In this configuration, the designed structure height was c=200μm, with a specified layer height of 100μm. As a result, two deposition passes were necessary to complete the fabrication. The specimens were collected after the first deposition pass, resulting in an expected height of 100μm. Filament consumption was approximately 0.09g per specimen, and the printing time was approximately 90s per specimen.

Figure 11 presents the results of the fourth and final configuration, displaying the CAD models (top), material-deposition paths (middle), and microscope images of the fabricated specimens (bottom) for four specimens, numbered 1 to 4 from left to right. In this configuration, the designed structure height was c=200μm, with a specified layer height of 100μm. Consequently, two deposition passes were necessary to complete the fabrication. The specimens were collected after the second deposition pass, resulting in an expected height of 200μm. The total filament consumption for Specimens 1 to 4 was approximately 0.12, 0.12, 0.12, and 0.13g, respectively, and the corresponding total printing times were 135, 136, 132, and 134s, respectively.

In summary, Figure 8 and Figure 9 show that neither single-layer configuration satisfactorily reproduces the designed pore geometry. Similarly, the specimens in Figure 10, which represent the first pass of the two-pass process, exhibit noticeable deviations from their CAD models. In contrast, Figure 11 shows a marked improvement after completion of the two-pass process. The following subsection provides a quantitative evaluation to substantiate these observations with numerical evidence.

### 4.3. Accuracy Evaluation

The accuracy of the fabricated porous structures was assessed from two perspectives: pore geometry and specimen height. Initially, cross-sectional images of both the designed and fabricated porous structures were compared to determine the fidelity of pore geometry reproduction. Subsequently, the heights of the fabricated specimens were measured and compared with their intended values to assess dimensional accuracy. These evaluations offer a quantitative assessment of the geometric fidelity of the fabricated porous structures.

#### 4.3.1. Pore Geometry Accuracy

Image processing is an effective approach for evaluating the geometric characteristics of fabricated structures, encompassing both porous and nonporous types. For instance, Okamoto and Ura [41] developed an image processing system to verify the geometric accuracy of 3D-printed objects by comparing images of designed and fabricated structures. This system generates binary images from CAD models and their fabricated counterparts, performs pixel-by-pixel comparisons, and identifies both matching and mismatched regions. The system also features an interface for generating reference images from CAD models and processing images of fabricated objects, with adjustable parameters for thresholding, noise reduction, masking, spatial scale, and image resolution. In a related study, Chawla et al. [42] examined various image-processing techniques to quantify porosity in additively manufactured polymer composites. Due to its demonstrated capabilities and ease of use, the system developed by Okamoto and Ura [41] was utilized in the present study to evaluate the geometric accuracy of fabricated PLA porous structures by comparing their cross-sectional images with corresponding reference images generated from CAD models.

Accordingly, let $B_{d}$ and $W_{d}$ denote the numbers of black and white pixels, respectively, in the binary image of the designed structure. Similarly, $B_{f}$ and $W_{f}$ denote the numbers of black and white pixels, respectively, in the binary image of the fabricated structure. In the comparison image, $B_{c}$ denotes the number of pixels that are black in both images, whereas $W_{c}$ denotes the number of pixels that are white in both images. The variable $Y_{c}$ denotes the number of pixels that are black in the designed image but white in the fabricated image, thus representing missing pore regions. The variable $V_{c}$ denotes the number of pixels that are white in the designed image but black in the fabricated image, and thus represents excess pore regions. Since all three images have the same dimensions and resolution, their total pixel counts satisfy $B_{d}+W_{d}=B_{f}+W_{f}=B_{c}+W_{c}+Y_{c}+V_{c}$.

In binary classification, a true positive (TP) is a positive element that is correctly classified as positive, whereas a true negative (TN) is a negative element that is correctly classified as negative. A false positive (FP) is a negative element that is incorrectly classified as positive, whereas a false negative (FN) is a positive element that is incorrectly classified as negative. In this study, black pore pixels were defined as the positive class, whereas white non-pore pixels were defined as the negative class. Therefore, the classification outcomes are related to the pixel counts in the comparison image as

(27) $$TP=B_{c},\;TN=W_{c},\;FP=V_{c},\;FN=Y_{c}.$$

Thus, in Equation (27), TP and TN represent correctly reproduced pore and non-pore pixels, respectively, whereas FP and FN represent excess and missing pore pixels, respectively. Consequently, several measures can quantify agreement between binary images. In this study, four measures were used: pore-region intersection over union (${\mathrm{IoU}}_{\mathrm{pore}}$), also known as the pore-region Jaccard index ($J_{\mathrm{pore}}$); precision (*P*); recall (*R*); and the $F_{1}$ score. The pore-region intersection over union, precision, recall, and $F_{1}$ score are defined by Equations (28), (29), (30), and (31), respectively.

(28) $$J_{\mathrm{pore}}={\mathrm{IoU}}_{\mathrm{pore}}=\frac{\mathrm{TP}}{\mathrm{TP}+\mathrm{FP}+\mathrm{FN}}=\frac{B_{c}}{B_{c}+V_{c}+Y_{c}}.$$

(29) $$P=\frac{\mathrm{TP}}{\mathrm{TP}+\mathrm{FP}}=\frac{B_{c}}{B_{c}+V_{c}}.$$

(30) $$R=\frac{\mathrm{TP}}{\mathrm{TP}+\mathrm{FN}}=\frac{B_{c}}{B_{c}+Y_{c}}.$$

(31) $$F_{1}=\frac{2PR}{P+R}=\frac{2\mathrm{TP}}{2\mathrm{TP}+\mathrm{FP}+\mathrm{FN}}=\frac{2B_{c}}{2B_{c}+V_{c}+Y_{c}}.$$

In this study, Equation (28) represents the pore-region intersection over union (${\mathrm{IoU}}_{\mathrm{pore}}$), also known as the pore-region Jaccard index ($J_{\mathrm{pore}}$). It quantifies the spatial overlap between the pore regions in the designed and fabricated structures. It accounts for both missing and excess pore regions and was therefore selected as the principal measure of geometric fidelity. Equation (29) represents precision (*P*), which indicates the proportion of pore pixels in the fabricated structure that correspond to the designed pore regions. A low precision value indicates a large proportion of excess pore pixels. Equation (30) represents recall (*R*), which indicates the proportion of designed pore pixels that were reproduced in the fabricated structure. A low recall value indicates a large proportion of missing pore pixels. Equation (31) represents the $F_{1}$ score, which combines precision and recall using their harmonic mean and quantifies the balance between excess and missing pore regions. All four measures range from zero to one. A value closer to one indicates better agreement between the designed and fabricated pore structures.

**Figure 12:**
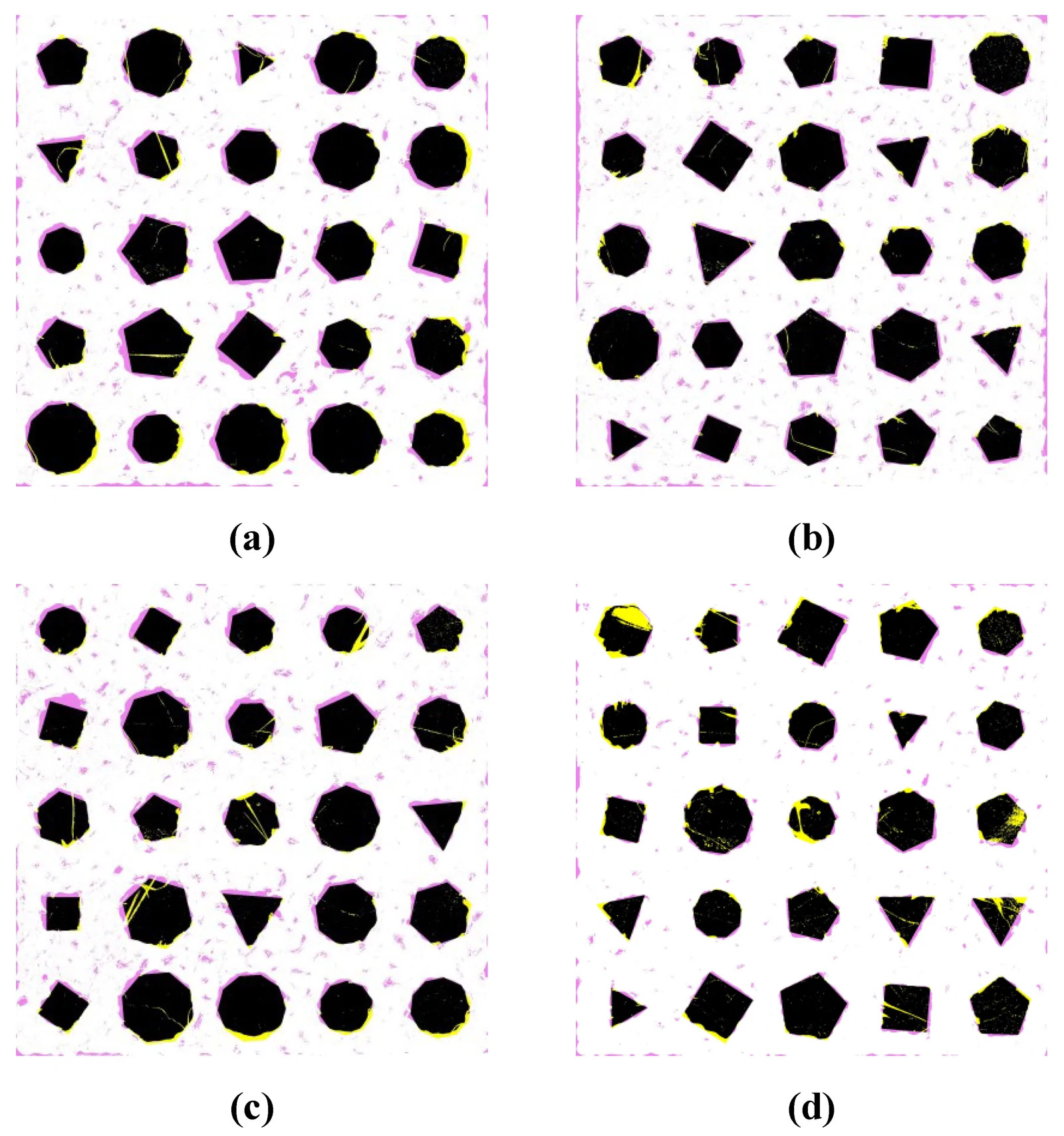
Comparison images of the four specimens shown in Figure 8: (**a**) the first specimen, (**b**) the second specimen, (**c**) the third specimen, and (**d**) the fourth (last) specimen, arranged from left to right. Black represents correctly reproduced pore regions, white represents correctly reproduced non-pore regions, violet indicates excess pore regions in the fabricated structure relative to the designed structure, and yellow indicates missing pore regions in the fabricated structure relative to the designed structure.

The comparison images of the four specimens shown in Figure 8 are presented in Figure 12. Table 4 summarizes the evaluation results for these four specimens. The pore-region *IoU* ranged from 73.17% to 79.38%, with an average of 76.77%. Precision ranged from 77.52% to 81.83%, with an average of 80.29%. Recall ranged from 92.87% to 96.36%, with an average of 94.56%. The $F_{1}$ score ranged from 84.51% to 88.50%, with an average of 86.84%.

**Figure 13:**
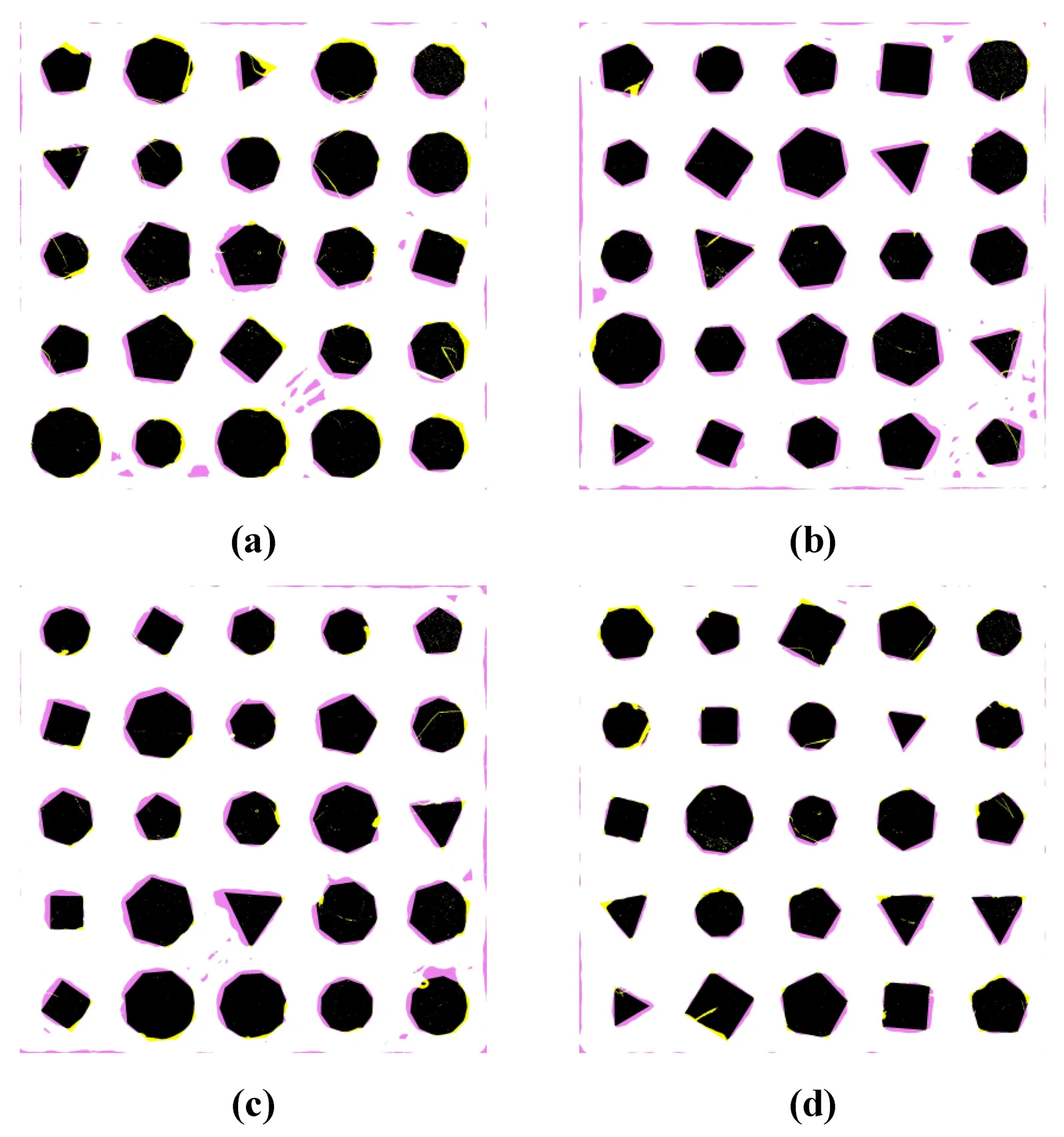
Comparison images of the four specimens shown in Figure 9: (**a**) the first specimen, (**b**) the second specimen, (**c**) the third specimen, and (**d**) the fourth (last) specimen, arranged from left to right. Black represents correctly reproduced pore regions, white represents correctly reproduced non-pore regions, violet indicates excess pore regions in the fabricated structure relative to the designed structure, and yellow indicates missing pore regions in the fabricated structure relative to the designed structure.

The comparison images of the four specimens shown in Figure 9 are presented in Figure 13. Table 5 summarizes the evaluation results for the four specimens in Figure 9. The pore-region *IoU* ranged from 77.83% to 85.75%, with an average of 81.24%. Precision ranged from 78.71% to 88.99%, with an average of 83.83%. Recall ranged from 94.28% to 98.58%, with an average of 96.46%. The $F_{1}$ score ranged from 87.53% to 92.33%, with an average of 89.62%.

**Figure 14:**
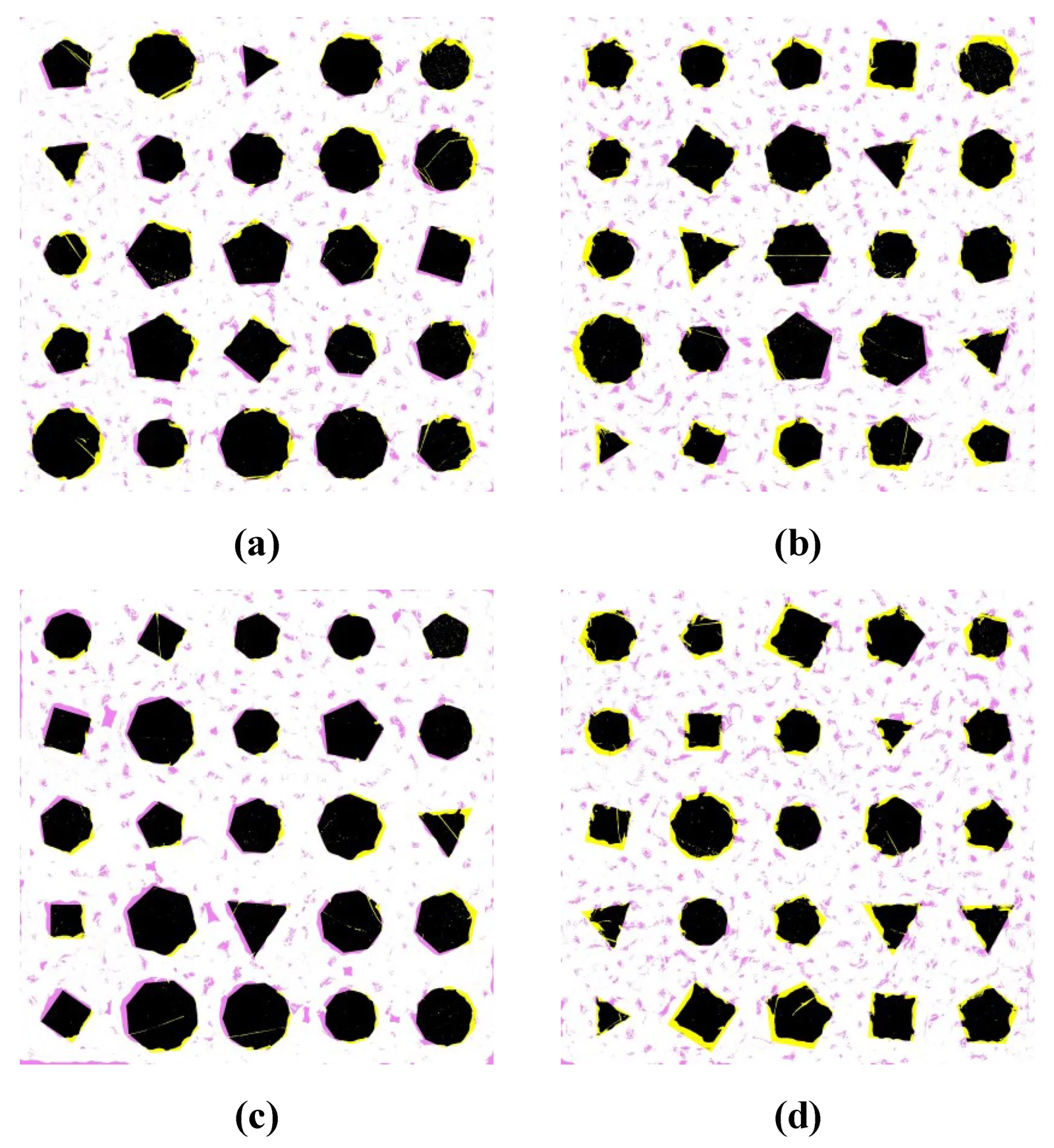
Comparison images of the four specimens shown in Figure 10: (**a**) the first specimen, (**b**) the second specimen, (**c**) the third specimen, and (**d**) the fourth (last) specimen, arranged from left to right. Black represents correctly reproduced pore regions, white represents correctly reproduced non-pore regions, violet indicates excess pore regions in the fabricated structure relative to the designed structure, and yellow indicates missing pore regions in the fabricated structure relative to the designed structure.

The comparison images of the four specimens shown in Figure 10 are presented in Figure 14. Table 6 summarizes the evaluation results for the four specimens in Figure 10. The pore-region *IoU* ranged from 64.41% to 78.33%, with an average of 72.75%. Precision ranged from 75.83% to 84.92%, with an average of 80.59%. Recall ranged from 81.05% to 94.28%, with an average of 88.02%. The $F_{1}$ score ranged from 78.35% to 87.85%, with an average of 84.11%.

**Figure 15:**
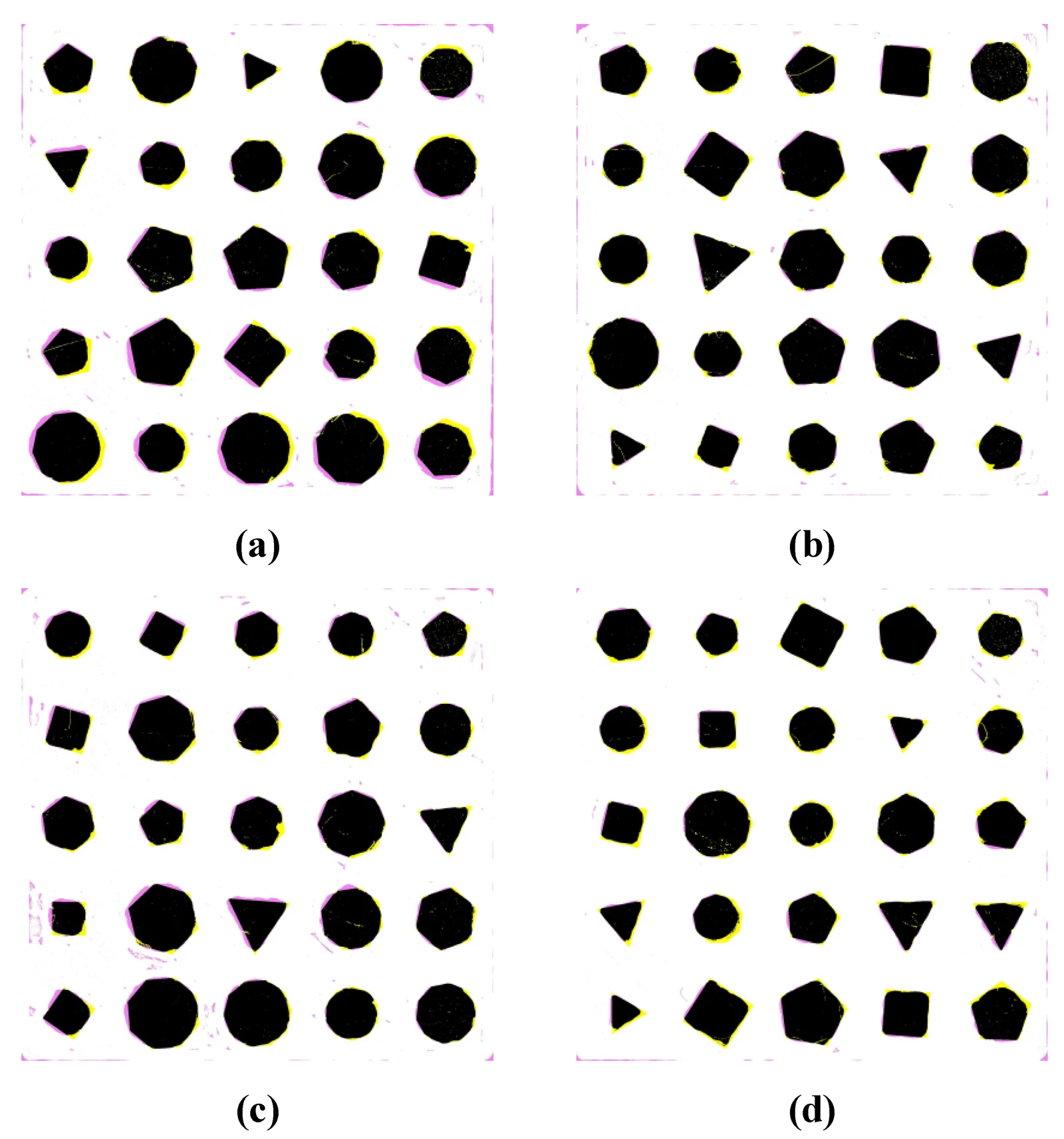
Comparison images of the four specimens shown in Figure 11: (**a**) the first specimen, (**b**) the second specimen, (**c**) the third specimen, and (**d**) the fourth (last) specimen, arranged from left to right. Black represents correctly reproduced pore regions, white represents correctly reproduced non-pore regions, violet indicates excess pore regions in the fabricated structure relative to the designed structure, and yellow indicates missing pore regions in the fabricated structure relative to the designed structure.

The comparison images of the four specimens shown in Figure 11 are presented in Figure 15. Table 7 summarizes the evaluation results for the four specimens in Figure 11. The pore-region *IoU* ranged from 84.52% to 90.08%, with an average of 87.56%. Precision ranged from 91.08% to 95.75%, with an average of 93.75%. Recall ranged from 91.46% to 94.44%, with an average of 93.00%. The $F_{1}$ score ranged from 91.61% to 94.78%, with an average of 93.36%.

**Figure 16:**
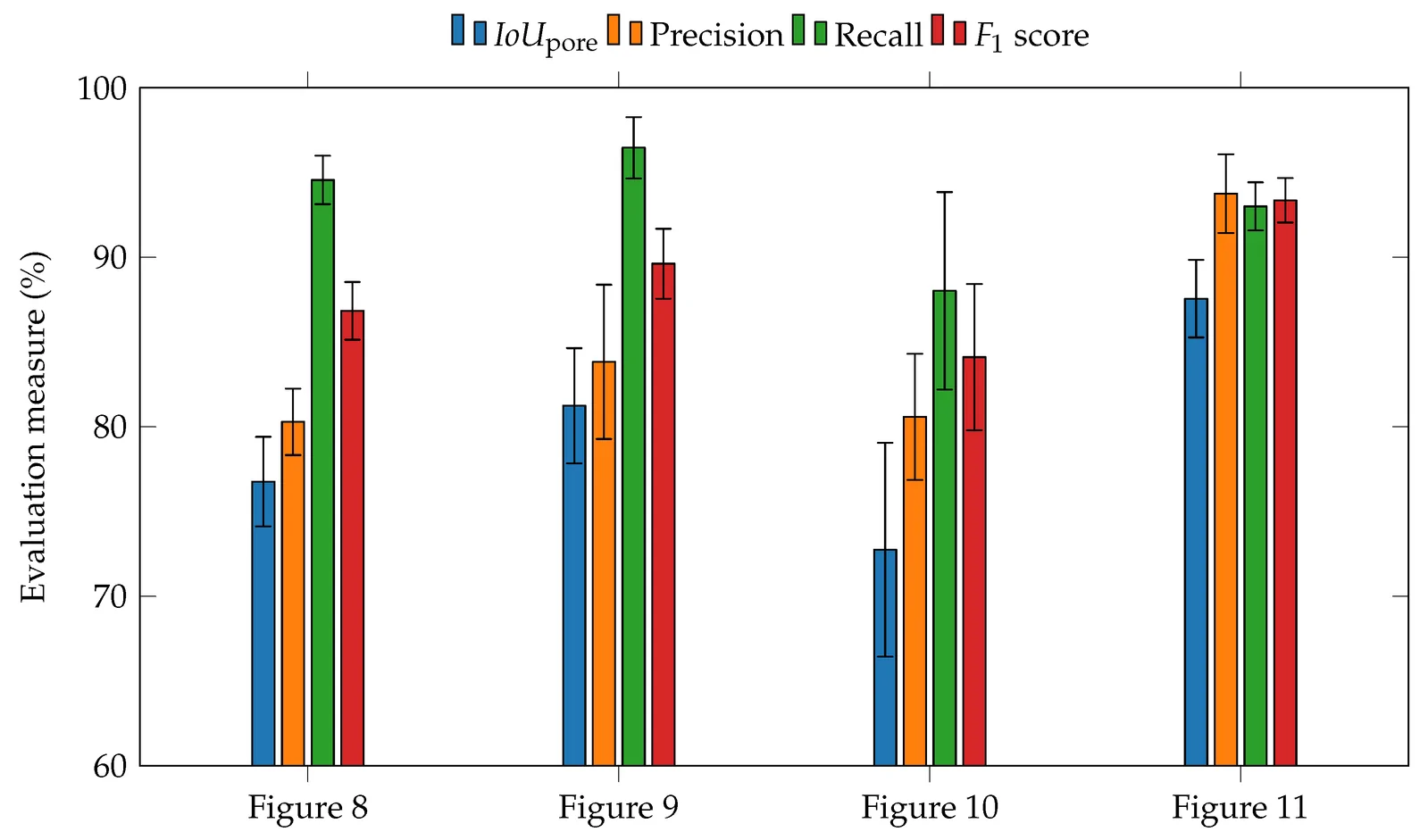
Comparison of the average pore-region intersection over union, precision, recall, and $F_{1}$ score for Figure 8, Figure 9, Figure 10 and Figure 11. The error bars indicate the deviation of the four specimens in each figure.

Figure 16 compares the average accuracy measures of the specimens in Figure 8, Figure 9, Figure 10 and Figure 11, along with their variability. The specimens in Figure 11 achieved the highest average *IoU* (87.56%), precision (93.75%), and $F_{1}$ score (93.36%), whereas those in Figure 9 exhibited the highest average recall (96.46%). The specimens in Figure 10 showed the lowest average *IoU* (72.75%) and the largest variability, particularly in *IoU*, recall, and $F_{1}$ score. In contrast, the specimens in Figure 11 exhibited both high average accuracy and low variability, indicating more consistent geometric agreement among these specimens.

Thus, among the configurations examined, the two-pass process with a model thickness of 200μm and a layer thickness of 100μm achieved the highest average geometric accuracy. These results indicate that layer thickness and deposition strategy influence the geometric fidelity of thin stochastic porous PLA structures.

#### 4.3.2. Porosity Accuracy

**Figure 17:**
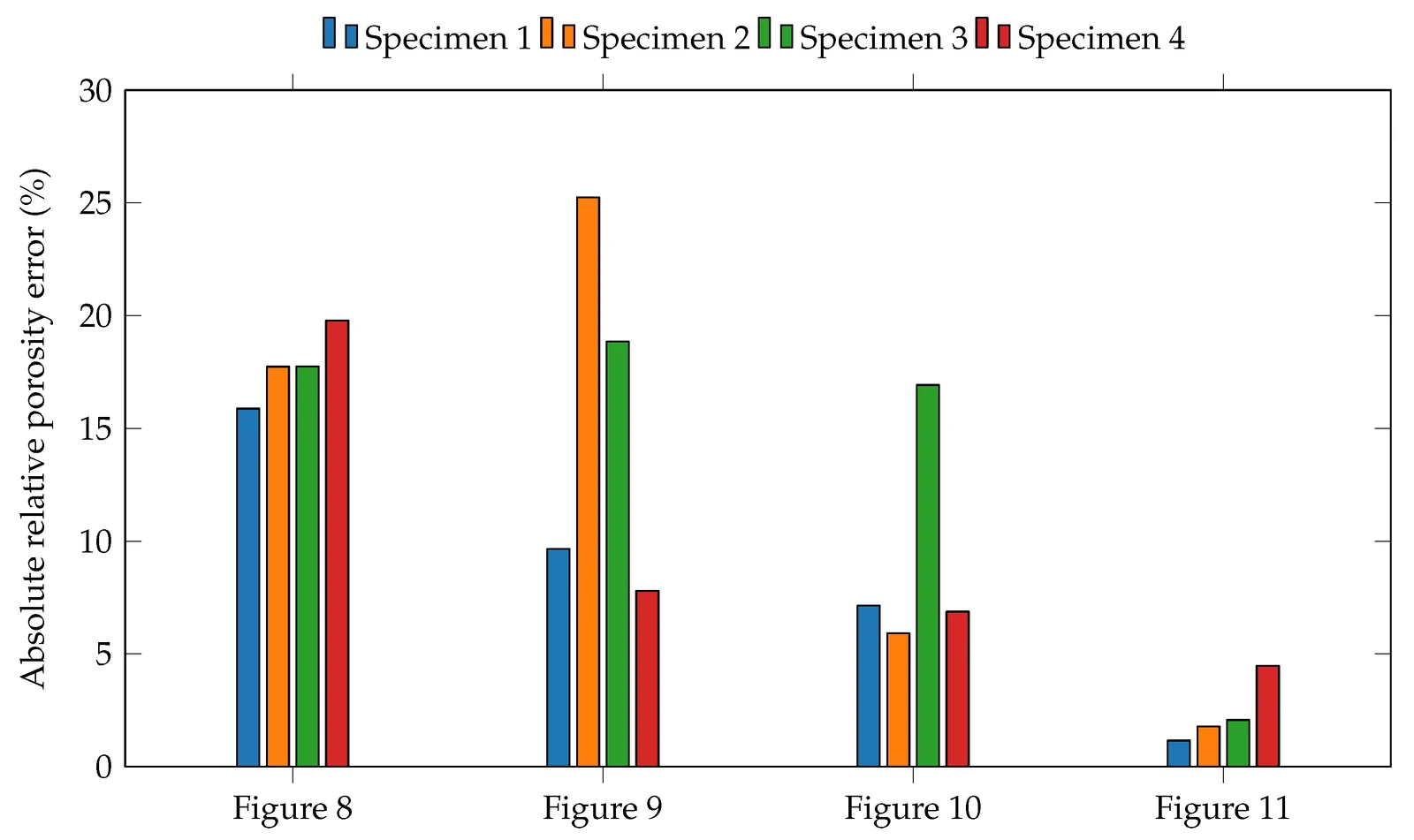
Comparison of the absolute relative porosity errors of the four specimens corresponding to Figure 8, Figure 9, Figure 10 and Figure 11. Blue, orange, green, and red bars represent the first, second, third, and fourth specimens, respectively. The errors were calculated from the absolute differences between the designed and fabricated porosity values relative to the designed porosity.

Table 8 summarizes the designed and real porosity values for the sixteen specimens corresponding to the four fabrication configurations. The designed porosity was calculated using the mathematical formulation described in Section 3, whereas the real (fabricated) porosity was determined from the binary images of the respective specimens by dividing the number of black pixels by the total number of pixels. As shown in Table 8, the real porosity was greater than the designed porosity in most cases. To quantify these deviations, the absolute relative porosity error was calculated as the absolute difference between the real and designed porosity values divided by the designed porosity, expressed as a percentage:

(32) $$E_{p}=\frac{\left|P_{f}-P_{d}\right|}{P_{d}}\times 100\%,$$

where $P_{f}$ and $P_{d}$ represent the fabricated and designed porosity, respectively. A smaller value of $E_{p}$ indicates better agreement between the fabricated and designed porosity, with zero indicating perfect agreement. Figure 17 compares the absolute relative porosity errors for the sixteen specimens. The average errors for Figure 8, Figure 9, Figure 10 and Figure 11 were 17.79%, 15.39%, 9.22%, and 2.38%, respectively. Among the four configurations, the specimens shown in Figure 11 exhibited the smallest average porosity error. These results indicate that the two-pass fabrication configuration with a specified layer height of 100μm provided better porosity accuracy for structures with a designed thickness of 200μm under the investigated fabrication conditions.

#### 4.3.3. Thickness Accuracy

**Figure 18:**
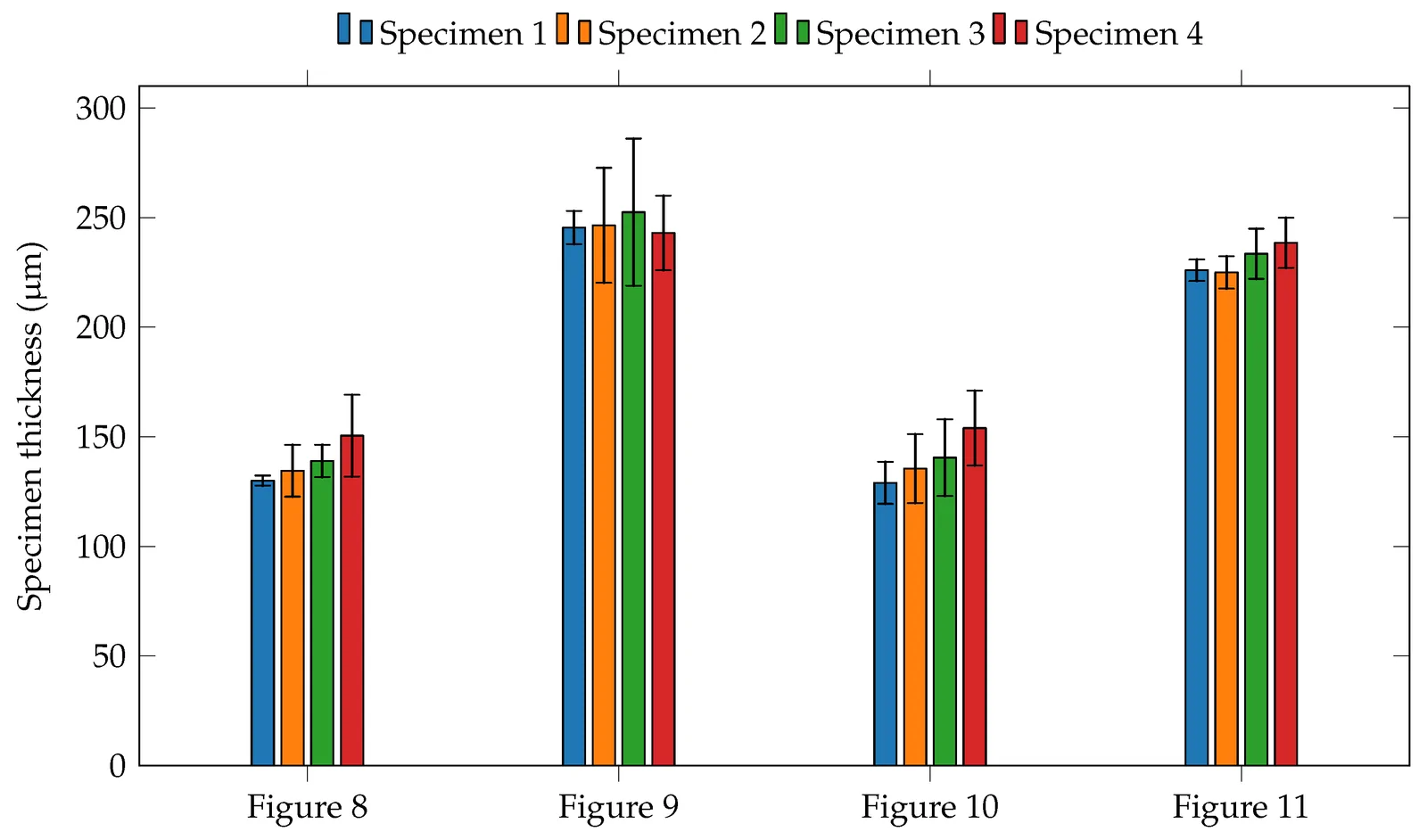
Comparison of the average specimen thicknesses for Figure 8, Figure 9, Figure 10 and Figure 11. Blue, orange, green, and red bars represent the first, second, third, and fourth specimens, respectively.

To evaluate thickness accuracy, each of the four sides of a specimen was measured five times using a digital Vernier caliper with a resolution of 1μm, yielding a total of 20 measurements per specimen. The average of these 20 measurements was considered the representative thickness of the specimen. Figure 18 compares the average thicknesses of the sixteen specimens corresponding to the four fabrication configurations. The error bars represent the standard deviation of the average thicknesses measured on the four sides of each specimen.

First, consider the specimens shown in Figure 8 and Figure 10, both of which were expected to have a fabricated thickness of 100μm. For Figure 8, the average thicknesses of Specimens 1–4 were 134, 141, 138, and 142μm, respectively. The overall average thickness was 139μm, corresponding to a deviation of approximately 39% from the expected thickness. Similarly, for Figure 10, the average thicknesses of Specimens 1–4 were 133, 140, 148, and 139μm, respectively. The overall average thickness was 140μm, corresponding to a deviation of approximately 40%. Both configurations produced specimens thicker than the expected 100μm, with comparable overall deviations.

Next, consider the specimens shown in Figure 9 and Figure 11, both of which were expected to have a fabricated thickness of 200μm. For Figure 9, the average thicknesses of Specimens 1–4 were 233, 267, 249, and 239μm, respectively. The overall average thickness was 247μm, corresponding to a deviation of approximately 23% from the expected thickness. Similarly, for Figure 11, the average thicknesses of Specimens 1–4 were 225, 226, 242, and 230μm, respectively. The overall average thickness was 231μm, corresponding to a deviation of approximately 15%. Although both configurations produced specimens thicker than expected, the two-pass configuration exhibited a smaller relative thickness deviation.

Overall, a model thickness of 200μm fabricated using two deposition passes of 100μm each exhibited the smallest relative thickness deviation.

## 5. Discussions

### 5.1. Variations in Pore Design

The proposed method provides a basis for the design and fabrication of thin structures with stochastic porosity. However, several geometric variations remain unexplored. In the present method, each pore is located at the center of its respective cell. The pore position can also be selected randomly within the cell, subject to geometric constraints. Such variations could produce different spatial distributions of pores. The polygonal pores in this study have equal edge lengths. Individual edge lengths could also vary to produce irregular polygonal pores. This modification would provide additional control over pore geometry. The proposed method uses extrusion to create through-holes. Other geometric operations, such as loft and sweep, could produce holes with variable cross-sectional shapes and orientations through the thickness of a component. The present method also uses cells with fixed dimensions. Random variations in cell dimensions could introduce additional variation in pore size and spatial distribution. However, such variations would require suitable constraints to maintain valid cell boundaries and avoid unintended intersections between adjacent pores. These geometric variations could extend the design space of stochastic porous structures. Their effects on printability, dimensional accuracy, and structural properties require further investigation.

### 5.2. Nested Porous Structures

**Figure 19:**
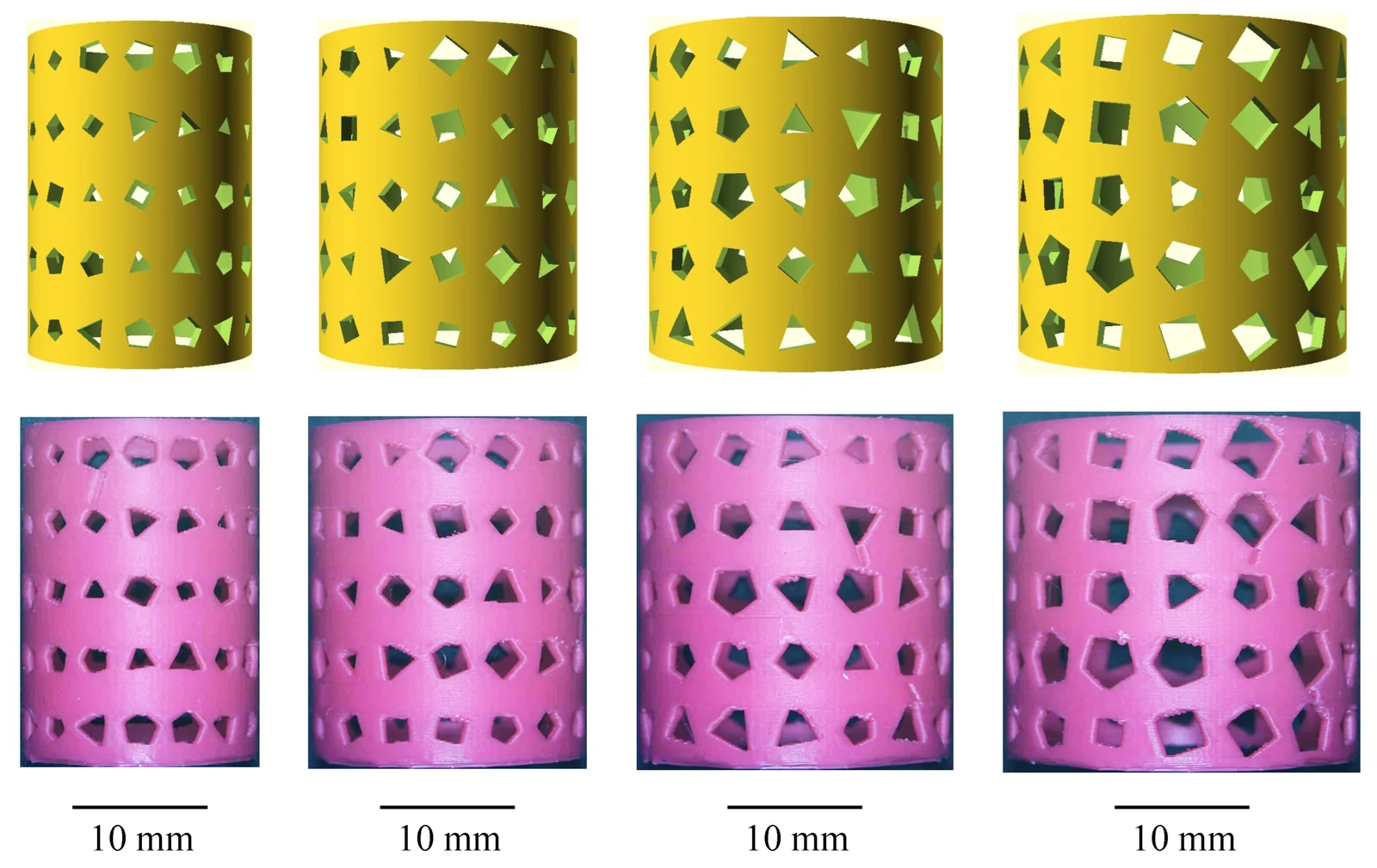
Extension of the proposed pore-generation method to a cylindrical shell.

**Figure 20:**
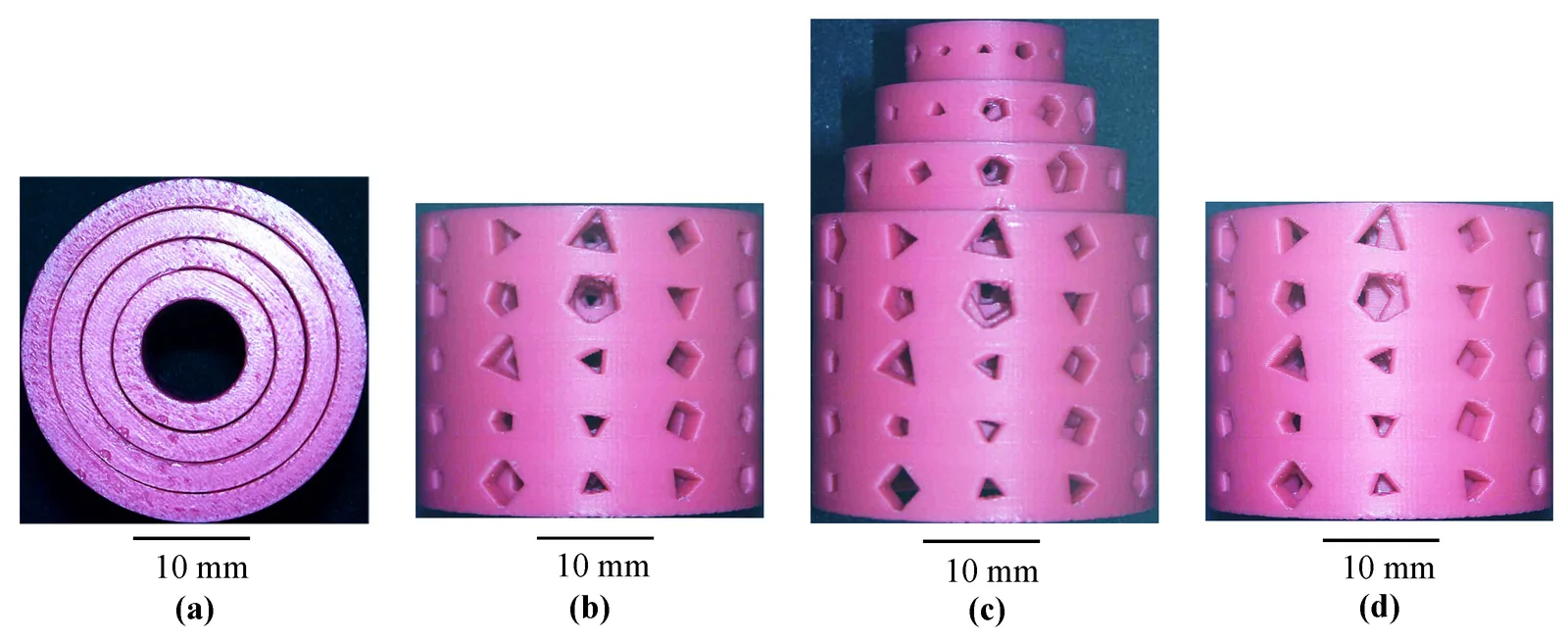
Concept of a nested porous cylindrical structure: (**a**) top view of four concentric porous cylindrical shells; (**b**) side view of the nested structure; (**c**) first relative angular orientation of the shells; and (**d**) second relative angular orientation of the shells.

The present study considers planar plates as the basis for pore generation. The proposed algorithm could be extended to curved surfaces through modifications to the cell definition and pore-generation procedures. For example, Figure 19 illustrates the generation of pores on a cylindrical shell. This extension also permits the construction of nested porous structures. Several porous cylindrical shells can be arranged concentrically to form a multilayer structure. Each shell can rotate relative to the others. Consequently, the overlap between pores in adjacent shells changes with their relative angular orientations. The two orientations produce different degrees of pore overlap, as illustrated in Figure 20c,d. The effective opening, therefore, depends on the relative angular positions of the cylindrical shells. This approach could produce effective openings smaller than the individual pores that can be fabricated. The minimum feature dimensions of each shell must still satisfy the fabrication constraints. The achievable effective aperture also depends on geometric alignment, manufacturing accuracy, and assembly tolerances. The concept offers a potential means to control the effective porosity of a structure through relative rotation without modifications to the individual shells. However, the relationship between angular orientation, effective pore size, and transport properties remains to be investigated. Future work should quantify the projected pore-overlap area, effective pore diameter, and open-area fraction as functions of the relative angular orientations. Experimental validation is also required to determine the effects of manufacturing errors and assembly tolerances on the achievable pore geometry. Figure 20a shows the top view of four concentric porous cylindrical shells. Figure 20b shows the corresponding side view. Figure 20c,d illustrate two different relative angular orientations of the shells.

### 5.3. Advanced Materials

Recent advancements in material science have led to the development of new materials aimed at enhancing the effectiveness of additive manufacturing. Among these, nanoparticle-reinforced polymers present significant potential for improving mechanical and thermal properties. For instance, Erdoğdu et al. [43] reported notable enhancements in the mechanical and thermal characteristics of zinc oxide nanoparticle-reinforced photopolymers. These enhancements may be advantageous for porous structures, which often experience reduced mechanical strength due to thin walls and pores. Nevertheless, superior material properties do not automatically result in improved performance of porous structures. Specifically, the applicability of nanoparticle-reinforced polymers for fabricating thin stochastic porous structures, as well as their influence on pore geometry, printability, and structural integrity, has yet to be fully determined. Consequently, further research is required to evaluate the use of these advanced materials in the additive manufacturing of porous structures.

## 6. Conclusions

This study presented a method for the design and fabrication of thin PLA components with stochastic porosity. The concluding remarks of this study are as follows.

(1) The proposed method divides a planar region into cells and generates polygonal pores within the respective cells. Random variations in pore geometry and orientation produce stochastic porous structures. The proposed method also avoids disconnected regions and excessively thin walls in the CAD models.
(2) The results of the three evaluations—pore geometry accuracy, porosity accuracy, and thickness accuracy—demonstrate that fabrication configuration significantly affects the geometric fidelity of thin stochastic porous PLA structures. Of the four configurations assessed, a designed thickness of 200μm produced with two deposition passes of 100μm each achieved the highest average pore geometry accuracy, the lowest average porosity error, and the smallest relative thickness deviation. Consequently, this configuration is identified as the most suitable under the examined fabrication conditions.
(3) Sensitivity analysis indicated that the pore-size parameter $\alpha_{ij}$ had the greatest influence on porosity, followed by the polygon-side parameter $m_{ij}$. The interaction between these parameters had a relatively small effect. In contrast, the rotation angle $\beta_{ij}$ did not influence porosity because it changes only the pore orientation without affecting its area or volume.
(4) The experiments demonstrated that thin stochastic porous polylactic acid (PLA) structures can be fabricated using a commercially available, conventional fused filament fabrication (FFF) 3D printer equipped with a 0.4mm nozzle. These findings suggest that specialized additive manufacturing equipment is not required for the fabrication of such structures. Nevertheless, careful selection of deposition strategy and layer thickness is essential to ensure adequate dimensional and geometric fidelity.
(5) The proposed method can be extended by varying pore locations, polygon shapes, and cell sizes, or by applying it to curved surfaces and nested porous structures. Notably, the relative rotation of concentric porous cylindrical shells may enable effective pore openings smaller than the individual fabricated pores. Future research should investigate these variations and assess their impact on geometric accuracy, effective pore size, and overall structure.

## Figures and Tables

**Table 1:** Key findings, limitations, and research needs identified from the literature.

Aspect	Key Findings	Limitations and Research Needs	Representative Studies
Design methods	Periodic unit cells, TPMS, Voronoi tessellations, stochastic point clouds, spherical voids, and fractals have been used to generate porous structures.	Most methods focus on repeated unit cells, three-dimensional scaffolds, or strut-based lattices. Thin structures with controllable stochastic polygonal pores have received limited attention.	[18,20,27,35,36,37,38,40]
Design parameters	Pore size, porosity, seed-point density, strut thickness, wall thickness, and spatial gradation can control mechanical and functional properties. Fractal level, scaling, and rotation provide additional geometric design parameters.	Random variation can create excessively small pores, thin walls, or disconnected solid regions. Geometric constraints are required to preserve fabricability.	[21,22,23,24,40]
Fabrication problems	Reported defects include closed pores, incomplete deposition, surface roughness, unintended porosity, and differences in relative density.	These defects become critical in thin stochastic structures because local pore and wall dimensions vary throughout the geometry.	[19,27,31,34]
Accuracy assessment	Digital calipers, microscopy, image processing, X-ray micro-tomography, porosity measurements, and volume comparisons have been used to assess fabrication accuracy.	Some studies used only visual comparison or reported no unified measure of geometric agreement. A direct quantitative comparison of designed and fabricated pore regions is required.	[18,28,29,30,32,34]
Applications	Porous structures have been investigated for biomedical scaffolds, energy absorption, acoustic control, and lightweight components.	Most studies emphasize mechanical or functional performance. The design accuracy and fabricability of thin stochastic PLA structures remain insufficiently explored.	[22,24,25,33]

**Table 2:** Specifications of the 3D printer.

Item	Details
Printer	Creality K2 Plus
Printing Technology	Fused Filament Fabrication (FFF)
Filament Material	Polylactic Acid (PLA)
Build Volume [mm]	350×350×350
Nozzle Diameter [mm]	0.4
Filament Diameter [mm]	1.75
Layer Thickness [mm]	0.1 or 0.2
Support	Not applied
Infill Rate [%]	100
Slicing Software	Creality Print 6.0

**Table 3:** Specifications of the digital microscope.

Item	Details
Digital Microscope	KEYENCE VHX-5000
Lens	VH-ZST
Magnification	20×, 100×, 200×, 500×, 1000×, and 2000×
Image Sensor	1/1.8-inch CMOS image sensor
Effective Pixels	1600×1200 pixels
Frame Rate	50 F/s
Working Distance [mm]	15
Light Source	High-intensity LED
Monitor	23-inch color LCD monitor
Monitor Resolution	1920×1080 pixels
Image File Format	JPEG, TIFF

**Table 4:** Pixel counts and calculated accuracy measures for the four specimens in Figure 8.

Specimen	$B_{c}$	$W_{c}$	$Y_{c}$	$V_{c}$	${\mathrm{IoU}}_{\mathrm{pore}}$ (%)	*P* (%)	*R* (%)	$F_{1}$ (%)
1	901,898	2,175,190	52,779	204,409	77.81	81.52	94.47	87.52
2	797,943	2,329,040	30,160	177,133	79.38	81.83	96.36	88.50
3	784,550	2,311,633	45,399	192,694	76.72	80.28	94.53	86.83
4	666,500	2,423,390	51,148	193,238	73.17	77.52	92.87	84.51

**Table 5:** Pixel counts and calculated accuracy measures for the four specimens in Figure 9.

Specimen	$B_{c}$	$W_{c}$	$Y_{c}$	$V_{c}$	${\mathrm{IoU}}_{\mathrm{pore}}$ (%)	*P* (%)	*R* (%)	$F_{1}$ (%)
1	900,100	2,232,765	54,577	146,834	81.72	85.97	94.28	89.94
2	816,350	2,285,365	11,753	220,808	77.83	78.71	98.58	87.53
3	805,326	2,323,304	24,623	181,023	79.66	81.65	97.03	88.68
4	688,434	2,531,426	29,214	85,202	85.75	88.99	95.93	92.33

**Table 6:** Pixel counts and calculated accuracy measures for the four specimens in Figure 10.

Specimen	$B_{c}$	$W_{c}$	$Y_{c}$	$V_{c}$	${\mathrm{IoU}}_{\mathrm{pore}}$ (%)	*P* (%)	*R* (%)	$F_{1}$ (%)
1	868,623	2,225,348	86,054	154,251	78.33	84.92	90.99	87.85
2	710,274	2,339,318	117,829	166,855	71.39	80.98	85.77	83.31
3	782,514	2,316,381	47,435	187,946	76.88	80.63	94.28	86.93
4	581,645	2,431,234	136,003	185,394	64.41	75.83	81.05	78.35

**Table 7:** Pixel counts and calculated accuracy measures for the four specimens in Figure 11.

Specimen	$B_{c}$	$W_{c}$	$Y_{c}$	$V_{c}$	${\mathrm{IoU}}_{\mathrm{pore}}$ (%)	*P* (%)	*R* (%)	$F_{1}$ (%)
1	879,682	2,293,452	74,995	86,147	84.52	91.08	92.14	91.61
2	777,878	2,470,733	50,225	35,440	90.08	95.64	93.93	94.78
3	783,841	2,441,053	46,108	63,274	87.75	92.53	94.44	93.48
4	656,378	2,587,483	61,270	29,145	87.89	95.75	91.46	93.56

**Table 8:** Comparison of the designed and real porosity of the sixteen specimens corresponding to the four fabrication configurations.

Figure	Specimen	Design Porosity (%)	Real Porosity (%)
Figure 8	1	28.63	33.18
	2	24.84	29.24
	3	24.89	29.31
	4	21.52	25.78
Figure 9	1	28.63	31.40
	2	24.84	31.11
	3	24.89	29.58
	4	21.52	23.20
Figure 10	1	28.63	30.68
	2	24.84	26.31
	3	24.89	29.11
	4	21.52	23.00
Figure 11	1	28.63	28.97
	2	24.84	24.39
	3	24.89	25.41
	4	21.52	20.56

## Data Availability

The data supporting the findings of this study are available from the corresponding author upon reasonable request.

## Supplementary Materials

The following supporting information can be downloaded at: https://www.mdpi.com/article/10.3390/polym18212708/s1, Listing S1: Complete OpenSCAD source code for generating a CAD model (STL data) of a stochastic porous structure.

## Abbreviations

The following abbreviations are used in this article:

3D	Three-dimensional
ABS	Acrylonitrile butadiene styrene
AM	Additive manufacturing
CAD	Computer-aided design
CMOS	Complementary metal-oxide-semiconductor
FDM	Fused deposition modeling
FFF	Fused filament fabrication
GO	Graphene oxide
IFS	Iterated function system
IoU	Intersection over union
JPEG	Joint Photographic Experts Group
LED	Light-emitting diode
PBAT	Poly(butylene adipate-co-terephthalate)
PHA	Polyhydroxyalkanoate
PLA	Polylactic acid
PVA	Polyvinyl alcohol
STL	Stereolithography mesh file format
TIFF	Tagged Image File Format
TPMS	Triply periodic minimal surface
