# Supplementary material for: On the Design and 3D Printing of Thin Stochastic Porous Structures Made of PLA
Source: Polymers (Basel). 2026 Nov 1;18(21):2708. doi: 10.3390/polym18212708 (PMC13765862; doi:10.3390/polym18212708)
Supplement: Supplementary file 1 [file polymers-18-02708-s001.zip › polymers-4606773-supplementary.pdf]

SUPPLEMENTARY MATERIAL S1

On the Design and 3D Printing of Thin Stochastic Porous Structures Made of PLA

Kazuki Sasaki[1] and Sharifu Ura[2,*]

[1] Graduate School of Engineering, Kitami Institute of Technology, Japan; d3247100056@std.kitami-it.ac.jp

[2] Division of Mechanical and Electrical Engineering, Kitami Institute of Technology, Japan; ullah@mail.kitami-it.ac.jp

[*] Correspondence: ullah@mail.kitami-it.ac.jp

S1. OpenSCAD Source Code for Stochastic Porous Structures

This supplementary document provides the OpenSCAD source code used to generate thin stochastic porous structures with polygonal through-holes. The code subdivides a square slab into a grid and assigns stochastic hole-size factors, polygon side counts, and rotation angles to individual grid cells. A Boolean subtraction produces the porous slab. The code also computes the theoretical average porosity from the generated geometry and reports a suggested STL filename. The listed parameters generate a CAD model with a slab side length of 20 mm and a structure height of 0.2 mm. Users can modify the geometric parameters and their ranges in accordance with Algorithm 1 to generate different porous structures. In addition, assigning a different integer value to `seed_base` changes the random-number sequence, allowing the generation of new stochastic realizations.

Listing S1: Complete OpenSCAD source code for generation of a stochastic porous slab.

```
//
// Porous slab with subtractive polygonal holes
// Grid-based stochastic geometric model
//

//==================================================
// User-defined parameters
//==================================================
a = 20;              // Slab side length [mm]
b = 0.2;             // Slab thickness [mm]
n = 5;               // Number of grid divisions per side

alpha_min = 0.5;     // Minimum hole-size factor
alpha_max = 0.8;     // Maximum hole-size factor

m_min = 3;           // Minimum number of polygon sides
m_max = 10;          // Maximum number of polygon sides

beta_min = 0;        // Minimum rotation angle [deg]
beta_max = 45;       // Maximum rotation angle [deg]

seed_base = 8;       // Base random seed
p = 1;               // Probability of generating a hole

//==================================================
```

```
// Derived parameters
//==================================================
d = a / n;          // Grid-cell side length
eps = 0.2;          // Extra extrusion depth for subtraction

echo("Grid spacing d =", d);

if (m_min < 3)
    echo("WARNING: m must be at least 3");

//==================================================
// Regular polygon vertices
//==================================================
function regular_polygon_points(radius, sides, rot_deg = 0) =
    [for (k = [0 : sides - 1])
        [radius * cos(rot_deg + 360 * k / sides),
         radius * sin(rot_deg + 360 * k / sides)]
    ];

//==================================================
// Theoretical porosity contribution of one cell
//==================================================
function pore_ratio(alpha, m) =
    m * alpha * alpha / 4 * sin(180 / m) * cos(180 / m);

function cell_porosity(i, j) =
    let(
        alpha = rands(alpha_min, alpha_max, 1,
            seed_base + 1000 * i + j)[0],
        m = floor(rands(m_min, m_max + 1, 1,
            seed_base + 5000 * i + 10 * j)[0]),
        rpore = rands(0, 1, 1,
            seed_base + 6000 * i + 60 * j)[0]
    )
    (rpore < p) ? pore_ratio(alpha, m) : 0;

function list_sum(v, i = 0) =
    (i >= len(v)) ? 0 : v[i] + list_sum(v, i + 1);

//==================================================
// One polygonal through-hole
//==================================================
module polygon_hole(cx, cy, thickness, radius, sides, rot_deg, rpore)
{
    if (rpore < p)
        translate([cx, cy, -eps / 2])
            linear_extrude(height = thickness + eps)
                polygon(
                    points = regular_polygon_points(
                        radius, sides, rot_deg
                    )
                );
}

//==================================================
// Generate all holes on the grid
//==================================================
module all_holes()
{
    for (i = [0 : n - 1])
    {
        for (j = [0 : n - 1])
        {
```

```
            x_ic = i * d + d / 2;
            y_jc = j * d + d / 2;

            alpha = rands(alpha_min, alpha_max, 1,
                seed_base + 1000 * i + j)[0];

            r = alpha * a / (2 * n);

            m = floor(rands(m_min, m_max + 1, 1,
                seed_base + 5000 * i + 10 * j)[0]);

            beta = rands(beta_min, beta_max, 1,
                seed_base + 9000 * i + 100 * j)[0];

            rpore = rands(0, 1, 1,
                seed_base + 6000 * i + 60 * j)[0];

            polygon_hole(x_ic, y_jc, b, r, m, beta, rpore);

            echo("Hole: m, alpha, radius, beta, p, rpore, porosity =",
                m, alpha, r, beta, p, rpore,
                (rpore < p) ? pore_ratio(alpha, m) : 0);
        }
    }
}

//==================================================
// Porous slab: slab minus polygonal through-holes
//==================================================
difference()
{
    cube([a, a, b], center = false);
    all_holes();
}

//==================================================
// Average theoretical cell porosity
//==================================================
PorosityList =
    [
        for (i = [0 : n - 1])
            for (j = [0 : n - 1])
                cell_porosity(i, j)
    ];

PorosityAverage = list_sum(PorosityList) / (n * n);

echo("PorosityAverage =", PorosityAverage);
echo("PorosityAverage (%) =", PorosityAverage * 100);

echo(str(
    "Suggested STL filename: PorousPlate_",
    a, "x", a, "x", b, "_",
    n, "x", n,
    "_A", alpha_min, "-", alpha_max,
    "_M", m_min, "-", m_max,
    "_R", beta_min, "-", beta_max,
    "_P", p,
    "_Seed", seed_base,
    "_Por", round(PorosityAverage * 1000000) / 10000,
    ".stl"
));
```
